# Macrophages trigger cardiomyocyte proliferation by increasing epicardial *vegfaa* expression during larval zebrafish heart regeneration

**DOI:** 10.1016/j.devcel.2022.05.014

**Published:** 2022-06-20

**Authors:** Finnius A. Bruton, Aryan Kaveh, Katherine M. Ross-Stewart, Gianfranco Matrone, Magdalena E.M. Oremek, Emmanouil G. Solomonidis, Carl S. Tucker, John J. Mullins, Christopher D. Lucas, Mairi Brittan, Jonathan M. Taylor, Adriano G. Rossi, Martin A. Denvir

**Affiliations:** 1 Centre for Cardiovascular Science, Queen’s Medical Research Institute, University of Edinburgh, Edinburgh EH16 4TJ, UK; 2 Centre for Inflammation Research, Queen’s Medical Research Institute, University of Edinburgh, Edinburgh EH16 4TJ, UK; 3 Department of Physics, University of Glasgow, Glasgow G12 8QQ, UK; 4 Brigham and Women’s Hospital, Harvard Medical School, Boston, MA 02115, USA

**Keywords:** regeneration, zebrafish, heart, macrophages, epicardium, vegf, notch, repair

## Abstract

Cardiac injury leads to the loss of cardiomyocytes, which are rapidly replaced by the proliferation of the surviving cells in zebrafish, but not in mammals. In both the regenerative zebrafish and non-regenerative mammals, cardiac injury induces a sustained macrophage response. Macrophages are required for cardiomyocyte proliferation during zebrafish cardiac regeneration, but the mechanisms whereby macrophages facilitate this crucial process are fundamentally unknown. Using heartbeat-synchronized live imaging, RNA sequencing, and macrophage-null genotypes in the larval zebrafish cardiac injury model, we characterize macrophage function and reveal that these cells activate the epicardium, inducing cardiomyocyte proliferation. Mechanistically, macrophages are specifically recruited to the epicardial-myocardial niche, triggering the expansion of the epicardium, which upregulates *vegfaa* expression to induce cardiomyocyte proliferation. Our data suggest that epicardial Vegfaa augments a developmental cardiac growth pathway via increased endocardial notch signaling. The identification of this macrophage-dependent mechanism of cardiac regeneration highlights immunomodulation as a potential strategy for enhancing mammalian cardiac repair.

## Highlights

• Heart regeneration in larval zebrafish is characterized in detail
• Macrophage ablation blocks cardiomyocyte proliferation after cardiac injury
• Macrophages synapse with epicardial cells and promote their proliferation
• Epicardial Vegfaa drives cardiomyocyte proliferation during cardiac regeneration

## Graphical abstract

**Figure:**
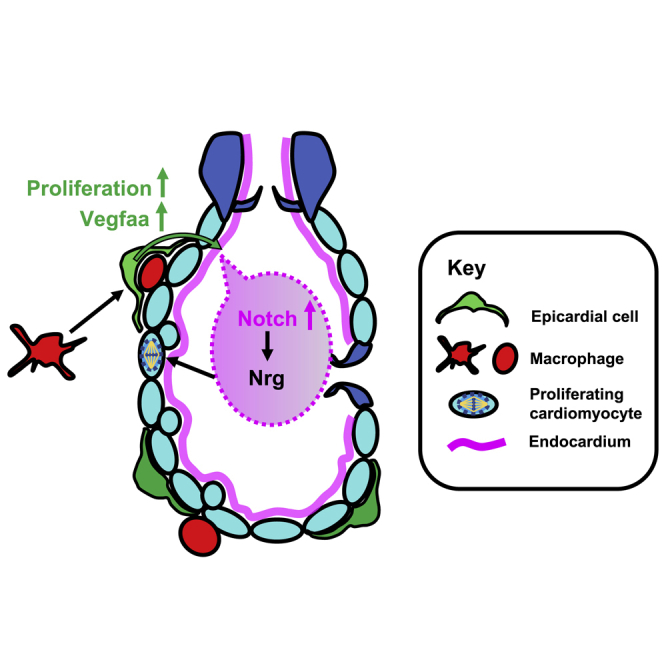

## Teaser

In this study, Bruton et al. test the requirement for macrophages for cardiac regeneration in larval zebrafish, finding macrophages to be necessary for cardiomyocyte proliferation via the activation of the epicardium. The identification of this macrophage-dependent mechanism of cardiac regeneration supports immunomodulation as a promising strategy for enhancing mammalian cardiac repair.

## Introduction

Zebrafish are highly regenerative, exhibiting the capacity to restore full structure and function to a wide range of tissues following injury (Becker et al., 2004; Sander et al., 2014; Ohnmacht et al., 2016; Basu Mallik et al., 2018; Bensimon-Brito et al., 2019). Cardiac injury is one such example where adult mammals are only able to facilitate maladaptive repair, whereas zebrafish exhibit full tissue regeneration (González-Rosa et al., 2011; Leuschner et al., 2012). In humans, the commonest and most severe form of cardiac injury is myocardial infarction (MI), where occlusion of a coronary artery triggers ischemic injury to the myocardium, leading to the loss of approximately 1 billion cardiomyocytes (Murry et al., 2006). Adult mammalian cardiomyocytes are considered largely post-mitotic, switching to hypertrophic growth shortly after birth and are therefore unable to restore lost myocardium, which is instead replaced with noncontractile scar tissue (Bergmann et al., 2015). Consequently, MI patients suffer sequelae of maladaptive remodeling, leading to left ventricular dilation and thinning of the scar, further decreasing the function of the heart (Pfeffer and Braunwald, 1990; Richardson et al., 2015). Hence, there is a need for medical innovation that can reverse or prevent this process.

In contrast to mammalian models of MI, zebrafish show full regeneration of lost myocardium via the dedifferentiation and proliferation of surviving cardiomyocytes (Jopling et al., 2010; Kikuchi et al., 2010). Cardiac regeneration is complex, with zebrafish hearts undergoing debridement of dead myocardium, followed by transient fibrosis, revascularization, and eventual replacement of cardiomyocytes (González-rosa et al., 2011). The inflammatory response has been demonstrated to be crucial for each of these key events, both in zebrafish and also in other regenerative species such as axolotls and neonatal mice (Aurora et al., 2014; Godwin et al., 2017; Lai et al., 2017). In particular, macrophages have emerged as important cellular regulators of tissue regeneration. Indeed, macrophage ablation has been shown to abrogate regeneration across multiple organs and organisms, including the adult zebrafish heart (Aurora et al., 2014; Godwin et al., 2017; Tsarouchas et al., 2018). However, the precise contribution of macrophages to cardiac repair has been complicated by disparate reports suggesting both beneficial and detrimental effects (Nahrendorf et al., 2007; Leuschner et al., 2012; Sager et al., 2016).

The larval zebrafish model of cardiac regeneration offers a tractable system to examine macrophage function in detail. Larval zebrafish regenerate more rapidly than adults, occurring in just 48 h after cardiac laser injury in 3-day-old larvae (Matrone et al., 2013; Kaveh et al., 2020). Combined with their amenability for live *in vivo* imaging and genetic tractability, this model represents a powerful tool with which to carefully examine how macrophages support multiple aspects of cardiac regeneration.

Here, we report an in-depth characterization of the macrophage response and several key regenerative processes in larval zebrafish cardiac regeneration, finding the heart regeneration program to be highly conserved between the larvae and adults. Abolition of the macrophage response demonstrated a requirement for these cells in removal of apoptotic cells, epicardial activation, and cardiomyocyte proliferation. We also demonstrate a mechanism by which macrophages exert their pro-proliferative effect, via epicardial Vegfaa and downstream endocardial notch signaling. Our study reveals that macrophages invade the epicardial-myocardial niche, inducing expansion of epicardial cell numbers that increases epicardial *vegfaa* expression, leading to an upregulation of endocardial notch signaling and consequently the cardiac developmental growth pathway.

## Results

### Macrophages display cellular heterogeneity following cardiac injury

**Figure 1:**
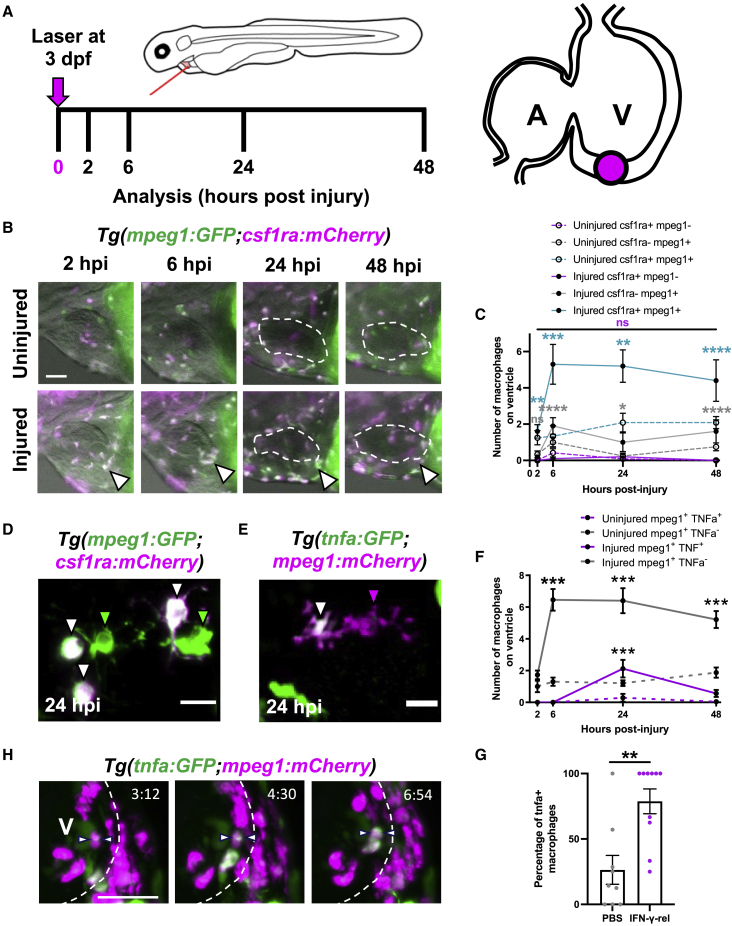
Cardiac macrophages display heterogeneity and plasticity following injury (A) Schematic illustrating the cardiac laser injury model, with imaging time points marked (left) and the injury site at ventricular apex of a 3 dpf larval heart marked (magenta circle) (right). (B) Representative lateral-view epifluorescence images of uninjured and injured hearts at the standard time points in *Tg(mpeg1:GFP;csf1ra:gal4:UAS:NfsB-mCherry)* (abbreviated to mpeg1:GFP;csf1ra:mCherry in all panels) illustrating macrophage heterogeneity; white arrow, ventricular apex; dashed line, heart outline. (C) Quantification of the number of csf1ra+mpeg1−, csf1ra-mpeg1+, and csf1ra+mpeg1+ macrophages on the ventricle in uninjured and injured larvae at standard time points, n = 10–12. (D) Representative LSFM image of csf1ra−mpeg1+ and csf1ra+mpeg1+ macrophages of different morphologies. (E) Representative LSFM image of tnfa*+*mpeg1+ and tnfa−mpeg1+ macrophages. (F) Quantification of the number of tnfa*+*mpeg1+ and tnfa−mpeg1+ macrophages on the ventricle in uninjured and injured larvae at standard time points, n = 10–25. (G) Quantification of the percentage of tnfa+ macrophages at 24 hpi following injection with IFN-γ-rel or PBS, n = 10. (H) Time-lapse time points for injured *Tg(tnfa:GFP;mpeg1:mCherry)* ventricles imaged live in the larvae by heartbeat-synchronized LSFM microscopy illustrating macrophage plasticity. Timestamps indicated, dashed line, ventricle outline; arrows, macrophage converting to tnfa*+*. Scale bars, 50 μm in (B) and (H) and 10 μm in (D) and (E). ^∗∗^ p ≤ 0.01, ^∗∗∗^ p ≤ 0.001, ^∗∗∗∗^ p ≤ 0.0001 (C and F). Two-way ANOVA followed by Holm-Sidak’s post-hoc test and (G) t test. Data are represented as mean ± SEM.

We first assessed macrophage heterogeneity and recruitment dynamics following larval cardiac injury. We crossed the zebrafish pan-macrophage reporter line *Tg(mpeg1:GFP)* with *Tg(csf1ra:gal4;UAS:mCherry-NfsB)* (shortened here to csf1ra: mCherry) (Figure S1A, related to Figure 1). Csf1ra (colony stimulated factor 1 receptor) is a cytokine required for macrophage development and used as a macrophage reporter promoter in mammals (Hume et al., 2017).

Larval hearts were lasered at the ventricular apex at 72 h post-fertilization (hpf) and imaged at 2, 6, 24, and 48 h post injury (hpi) (Figure 1A). Macrophages migrate to the injured ventricular apex within 2 h, peak at 6 and maintain elevated numbers until 48 hpi (Figures 1B and 1C). We found that not all recruited macrophages were co-positive for both transgenes, resulting in three subsets (1) mpeg1+csf1ra− (19.3% ± 5.1%), (2) mpeg1−csf1ra+ (2.8% ± 2.1%), and (3) mpeg1+csf1ra+ (77.9% ± 5.7%). Similar dynamics were seen for subsets 1 and 3, but since mpeg1−csf1ra+ were exceedingly rare, it is not possible to know if the dynamics are likewise similar. Both subsets exhibit a range of morphologies with no overt difference between groups (Figure 1D; Video S1). Importantly, our data demonstrate that larval macrophages recruited to cardiac injury are heterogeneous in their marker expression, similar to adult zebrafish (Bevan et al., 2020), and suggest a comparatively complex macrophage response in the larval model.

Video S1. LSFM-acquired heartbeat-synchronized time-lapse of a *Tg(csf1ra:mCherry;mpeg1:GFP)* heart showing macrophage heterogeneity following cardiac injury, related to Figure 1

### Macrophages display cellular plasticity following cardiac injury

To examine if macrophages display plasticity and convert to an inflammatory phenotype in the larval cardiac injury model, we performed cardiac laser injury on *Tg*(*tnf*a*:GFP;mpeg1:mCherry*) larvae (Figures 1E and 1F). Quantification of tnfa+ macrophage number revealed a transient tnfa+ subset (19.3% ± 4.9% of mpeg1+ macrophages, n = 24), found only at the 24 hpi time point and rarely in uninjured larvae (Figure 1F). Interestingly, csf1ra+ macrophages also become tnfa+ and do so more frequently (30.8% ± 8.1% of macrophages) (Figures S1B–S1D, related to Figure 1). We also observed that from 24 hpi, macrophages retract their pseudopods and become spherical, further suggesting a shift in phenotype (Figures S1E and S1F, related to Figure 1).

We reasoned that if tnfa+ macrophages were indeed inflammatory macrophages then application of M1-polarization cytokine IFN-γ would increase their abundance. A single intravenous injection of zebrafish recombinant protein IFN-γ-rel, immediately prior to cardiac injury, increased the proportion of tnfα+mpeg1+ macrophages from 26.4% ± 11.0% in PBS-injected controls to 78.8% ± 9.5%, supporting the suggestion that these were inflammatory macrophages (Figures 1G, S1G, and S1H).

Furthermore, *in vivo* imaging live in the beating heart showed recruited macrophages transitioning to tnfa:GFP+ after arrival at the injured ventricle, confirming that this represents true *in situ* conversion (Figure 1H; Video S2). Taken together, these data show that, as in adults, macrophages display plasticity and become inflammatory in response to larval cardiac injury.

Video S2. LSFM-acquired heartbeat-synchronized time-lapse of a *Tg(mpeg1:mCherry;tnfa:GFP)* heart showing macrophage plasticity following cardiac injury, related to Figure 1

### Larval cardiac laser lesions are similar in structure to adult cryoinjury

**Figure 2:**
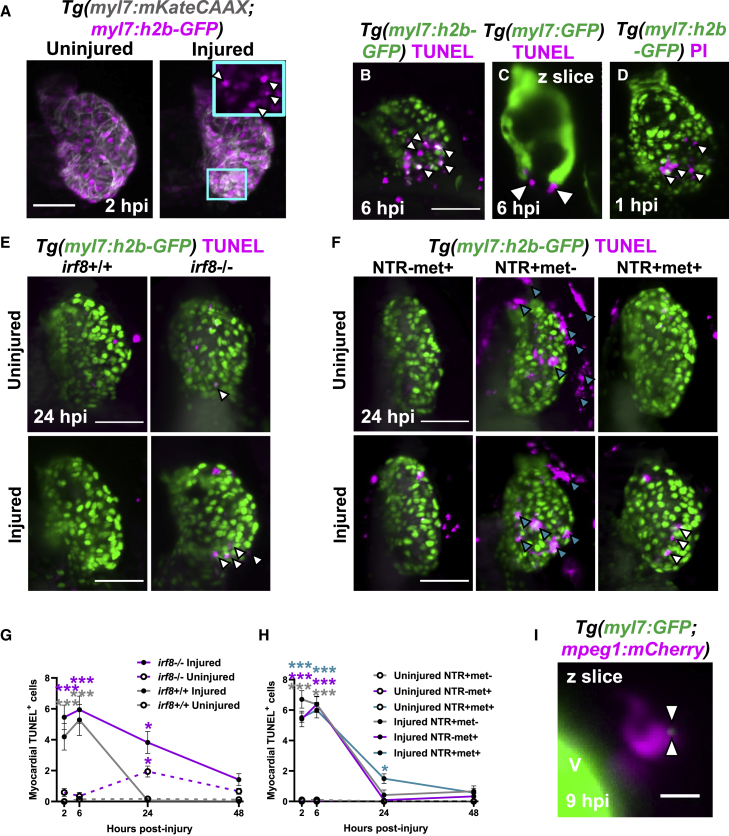
Macrophages are required for the timely removal of apoptotic cardiomyocytes (A–C) (A) Images of uninjured and injured *Tg(myl7:h2b-GFP;myl7:mKateCAAX)* ventricles. Cyan outlined zoom panel highlights condensed nuclei (white arrowheads). Images of TUNEL stained hearts 6 hpi in (B) *Tg(myl7:h2b-GFP)* and (C) *Tg(myl7:GFP)* larvae. White arrowheads, apoptotic cardiomyocytes/myocardium. (D) Image of a propidium iodide (PI)-stained *Tg(myl7:h2b-GFP)* heart at 1 hpi. White arrowheads, necrotic debris. (E) Images of uninjured and injured *irf8*^*+/+*^ and *irf8*^*−/−*^*Tg(myl7:h2b-GFP)* ventricles stained by TUNEL at 24 hpi. White arrowheads, TUNEL+ cells. (F) Images of uninjured and injured *Tg(myl7:h2b-GFP;csfr1a:NfsB-mCherry)* ventricles stained by TUNEL per macrophage ablation model injury group at 24 hpi. Cyan arrowheads, macrophages; white arrowheads, TUNEL+ cells. (G) TUNEL+ myocardial cells in uninjured and injured, *irf8*^*+/+*^ and *irf8*^*−/−*^*Tg(myl7:h2b-GFP)* ventricles, n = 15–29. (H) TUNEL+ myocardial cells in uninjured and injured *Tg(myl7:h2b-GFP;csfr1a:NfsB-mCherry)* ventricles per macrophage ablation group, n = 10–12. (I) z slice of LSFM-acquired z stack, at 9 hpi, showing internalized myocardial debris (white arrowheads) in a macrophage in a *Tg(myl7:GFP;mpeg1:mCherry)* larva, V, ventricle-surface. Scale bars, 50 μm for (A)–(F) and 10 μm for (I). All representative images are 3D LSFM shown as maximum intensity projections unless otherwise stated. ^∗^ p ≤ 0.05, ^∗∗^ p ≤ 0.01, ^∗∗∗^ p ≤ 0.001. Two-way ANOVA followed by Holm-Sidak’s post-hoc tests. Data are represented as mean ± SEM.

To validate analyses of macrophage function in the larval injury model, we next sought to determine if the laser lesion is comparable with adult cryoinjury and mammalian infarcts. Using the line *Tg(myl7:mKateCAAX;myl7:h2b-GFP)*, which labels cardiomyocyte sarcolemma and chromatin respectively, we observed that following injury, a circlet of cardiomyocytes with pyknotic nuclei formed (Figure 2A). These pyknotic nuclei were TUNEL+ at 6 hpi, confirming apoptosis, and they encircled the GFP-negative (−) lesion (Figures 2B and 2C). Heartbeat-synchronized light-sheet fluorescence microscopy (LSFM) (Taylor et al., 2019) showed that nuclear condensation occurred extremely rapidly, being identified by 1.5 hpi (Figure S2A; Video S3, related to Figure 2).

Video S3. LSFM-acquired heartbeat-synchronized time-lapse of a *Tg(myl7:h2b-GFP;myl7:mKateCAAX)* heart following cardiac injury showing cardiomyocyte apoptosis following injury, related to Figure 2

We hypothesized that the GFP− epicenter of the laser lesion may contain cells that immediately necrose upon injury. To assess this, we labeled necrotic cells with propidium iodide (PI) injected intravenously immediately following injury (<0.5 hpi). We found that there were indeed PI+ cells in the GFP− region, and PI+ debris scattered across the proximal myocardium from 1 hpi (Figure 2D). Time-lapse imaging of PI-injected, injured *Tg(mpeg1:GFP;myl7:h2b-GFP;myl7:mKateCAAX)* hearts showed that necrotic cells are rapidly cleared within the first 0–2 hpi (Figure S2B; Video S3, related to Figure 2). Necrotic cells either disintegrated or were squeezed out from the myocardium into the pericardial cavity, independently of macrophage contact (Video S4). Overall, this characterization confirms the structure of the laser lesion mirrors the necrotic infarct and apoptotic border zone observed in adult zebrafish cryoinjury and mammalian MI (Krijnen et al., 2002; González-rosa et al., 2011).

Video S4. LSFM-acquired heartbeat-synchronized time-lapse of a *Tg(myl7:h2b-GFP;mpeg1:GFP)* heart injected with propdium iodide showing PI+ cardiomyocyte expulsion following cardiac injury, related to Figure 2

### Macrophages contribute to the removal of apoptotic cardiomyocytes following injury

We next sought to understand what role macrophages play in the regeneration of the larval heart, which occurs within only 48 h of the initial injury (Matrone et al., 2013; Kaveh et al., 2020). We used two different methods to induce macrophage-less hearts. First, we used the *Tg(csf1ra:gal4;UAS:mCherry-NfsB)* line (abbreviated hereafter to csf1ra:NfsB-mCherry) that expresses a nitroreductase enzyme NfsB in macrophages, which induces cell-specific apoptosis when exposed to prodrug metronidazole (Pisharath et al., 2007) (Figures S3A–S3D, related to Figure 2). Macrophage ablation only occurs in larvae expressing the nitroreductase (NTR) and in the presence of metronidazole (NTR+met+). Therefore, larvae only expressing the nitroreductase (NTR+met−) or only in the presence of metronidazole (NTR-met+) are used as macrophage-replete control groups. The second method was the use of the macrophage-null *irf8*^*−/−*^ mutant (Shiau et al., 2015), IRF8 being a transcription factor required for macrophage development (Figures S3E–S3H, related to Figure 2).

To determine if macrophages are required for the removal of apoptotic cells, we performed TUNEL staining on *irf8*^*−/−*^ and *irf8*^*+/+*^ *Tg(myl7:h2b-GFP)* (Figures 2E and 2G). In injured irf8^+/+^ hearts, the number of apoptotic cardiomyocytes significantly increased at 2 and 6 hpi compared with uninjured controls (4.1 ± 0.9 versus 0.0 ± 0.0 and 5.3 ± 1.0 versus 0.1 ± 0.1, respectively, n = 15–29) but returned to baseline by 24 hpi. Although injured macrophage-null *irf8*^*−/−*^ hearts showed a similar initial pattern of cell death at 2 and 6 hpi (5.5 ± 0.8 and 5.9 ± 0.9 apoptotic cardiomyocytes, respectively), apoptotic cardiomyocyte cells were still present at 24 hpi, only returning to uninjured levels by 48 hpi.

In the macrophage ablation model, we saw a similar pattern of results where the numbers of apoptotic cells were negligible in uninjured hearts of all treatment groups but peaked at 6 hpi following injury (NTR+met−, NTR-met+ & NTR+met+ = 6.4 ± 0.5, 6.4 ± 0.5, and 6.0 ± 0.5, n = 10–12) (Figures 2F and 2H). By 24 hpi, the non-ablated groups no longer possessed significantly increased numbers of TUNEL+ myocardial cells; however, the macrophage-ablated group showed a retention of apoptotic cells at 24 hpi (NTR+met+ = 1.5 ± 0.3) that resolved by 48 hpi.

To verify that macrophages are directly removing myocardial debris, we performed time-lapse imaging of injured *Tg(myl7:GFP;mpeg1:mCherry)* larvae. We observed small GFP+ pieces of myocardial debris near the GFP− lesion being removed and internalized by macrophages (Figures 2I and S2C; Video S5), confirming the essential role of macrophages in lesion debridement.

Video S5. LSFM-acquired heartbeat-synchronized time-lapse of a *Tg(myl7:GFP;mpeg1:mCherry)* heart, 3D surface rendered, showing the removal and internalization of myocardial debris by macrophages following injury, related to Figure 2

### Macrophages are not obligatory for the structural or functional recovery of the larval heart

**Figure 3:**
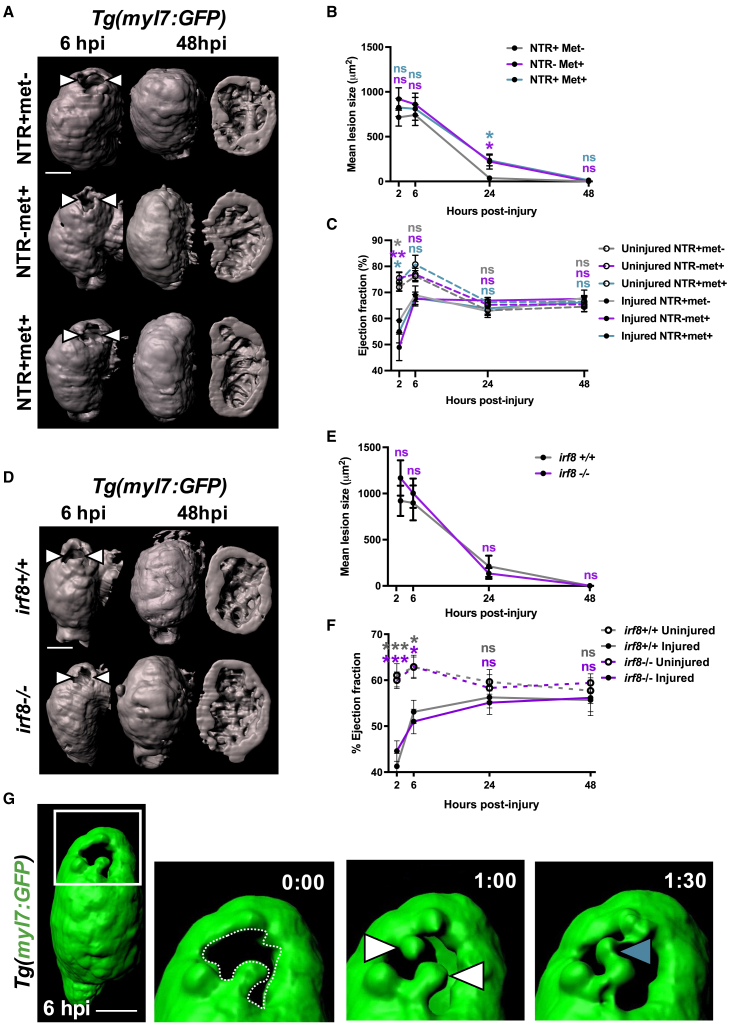
Macrophages not required for the recovery of cardiac structure or function (A) Representative GFP surface renders of LSFM z stacks of injured ventricles in *Tg(myl7:GFP;csfr1a:NfsB-mCherry)* larvae, macrophage ablation groups as indicated in the figure. Abluminal myocardial surface is shown at 6 hpi (left), and abluminal and luminal surfaces are shown at 48 hpi following regeneration (middle and right). White arrowheads, laser lesion. (B) Quantification of mean lesion size in injured *Tg(myl7:GFP;csfr1a:NfsB-mCherry)* larvae per macrophage ablation group, n = 11–22. (C) Quantification of ventricular ejection fraction in uninjured and injured *Tg(myl7:GFP;csfr1a:NfsB-mCherry)* larvae per macrophage ablation group, n = 10–12. (D) Representative GFP surface renders of LSFM-acquired z stacks of injured ventricles from *irf8*^*+/+*^ and *irf8*^*−/−*^*Tg(myl7:GFP)* larvae. Abluminal myocardial surface is shown at 6 hpi (left), and abluminal and luminal surfaces are shown at 48 hpi following regeneration (middle and right). White arrowheads, laser lesion. (E) Quantification of mean lesion size in injured *irf8*^*+/+*^ and *irf8*^*−/−*^*Tg(myl7:GFP)* larvae, n = 15. (F) Quantification of ventricular ejection fraction in uninjured and injured *irf8*^*+/+*^ and *irf8*^*−/−*^*Tg(myl7:GFP)* larvae n = 15–20. (G) Time-lapse time points of a GFP-surface-rendered, injured *Tg(myl7:GFP)* ventricle from 6 hpi. White box, zoom panel; white arrowheads, myocardial buds; cyan arrowhead, myocardial bridge. ^∗^ p ≤ 0.05, ^∗∗∗^ p ≤ 0.001. Two-way ANOVA followed by Holm-Sidak’s post-hoc tests. Scale bars, 50 μm. Data are represented as mean ± SEM.

Next, we sought to investigate if macrophages are required for structural and functional recovery of the larval heart following laser injury. We injured *Tg(myl7:GFP;csf1ra:NfsB-mCherry)* larvae following macrophage ablation and acquired serial 3D scans of the cardiac structure of individual larvae by heartbeat-synchronized LSFM (Figure 3A). In all treatment groups, the lesion size was consistent between 2 and 6 hpi, with no difference between groups. By 24 hpi, the lesion had almost completely regressed (95% to 37.1 μm^2^ ± 24.4, n = 11–22) in macrophage-replete NTR+met− larvae (Figure 3B). However, for larvae in the macrophage-ablated NTR+met+ and the other macrophage-replete NTR-met+ group, lesion closure was slightly delayed at 24 hpi (73% and 75% to 234.7 ± 59.7 μm^2^ and 221.4 ± 84.6 μm^2^, respectively). By 48 hpi, the lesions of larvae from each group had entirely regressed and luminal surface renders of injured ventricles showed normal trabecular structure (Figure 3A). These results suggest macrophages are not required for lesion closure but that metronidazole-treatment slightly delays this process.

Using *Tg(myl7:GFP)* larvae, we acquired lateral-view videos of beating hearts with epifluorescence microscopy and tested if macrophage ablation affected recovery of cardiac function. Immediately following injury at 2 hpi, volumetric ejection fraction was decreased in all groups from 74% in uninjured ventricles to 54% in injured (Figure 3C). Ejection fraction recovered quickly by 6 hpi in all treatment groups, (∼78% injured versus ∼87% uninjured) and by 24 and 48 hpi, injured hearts were functionally indistinguishable from uninjured hearts. These data suggest that injured larval hearts recover their function rapidly and that this recovery is not macrophage dependent.

We next performed identical experiments examining the recovery of cardiac structure and function with *Tg(myl7:GFP)* larvae on an *irf8* mutant background. Both *irf8*^*+/+*^ and *irf8*^*−/−*^ genotype larvae showed substantial lesion regression (∼80%) between 6 and 24 hpi (898.6 μm^2^ ± 189.7 to 211.6 μm^2^ ± 115.8 versus 1,002.9 μm^2^ ± 158.4 to 113.89 μm^2^ ± 59.5, respectively) (Figures 3D and 3E). No difference in lesion size was seen at any time point, and both genotypes had completely closed their lesions by 48 hpi. Normal trabecular structure was seen in both groups at 48 hpi following full structural recovery (Figure 3D). The recovery of ejection fraction in this model followed the same trend as that of the metronidazole-nitroreductase model, with the ejection fraction of injured larvae being indistinguishable from uninjured larvae by 24 hpi in both genotypes (Figure 3F). Our near identical findings in the *irf8* macrophage-null model confirm that larval hearts rapidly recover following laser injury and that this process is macrophage independent.

Finally, we wished to understand the mechanism of lesion closure. We performed heartbeat-synchronized time-lapse imaging of lesions in *Tg(myl7:GFP)* larvae immediately following injury. We observed GFP+ myocardial budding on opposite sides of the lesion border zone and subsequent invasion into the lesion, adhering to each other to form bridges (Figure 3G; Video S6). Repeating this experiment in *Tg(myl7:h2b-GFP;myl7:mKateCAAX)* larvae facilitated the tracking of individual cardiomyocytes by virtue of their labeled nuclei and plasma membranes (Video S7; Figure S2D, related to Figures 2 and 3). We found that cardiomyocytes bordering the lesion did not divide but extended protrusions into the lesion until they adhered with other single cardiomyocytes bridging from the opposing side of the lesion. These imaging insights suggest that myocardial structure is first restored by morphogenesis rather than cell division.

Video S6. LSFM-acquired heartbeat-synchronized time-lapse of a *Tg(myl7:GFP)* heart, 3D surface rendered, showing the budding and bridging of wound margin myocardium following injury, related to Figure 3

Video S7. LSFM-acquired heartbeat-synchronized time-lapse of a *Tg(myl7:h2b-GFP;myl7:mKateCAAX)* heart, showing the budding and bridging of individual wound-margin cardiomyocytes following injury, related to Figure 3

### Macrophage ablation abolishes an injury-associated increase in cardiomyocyte proliferation

**Figure 4:**
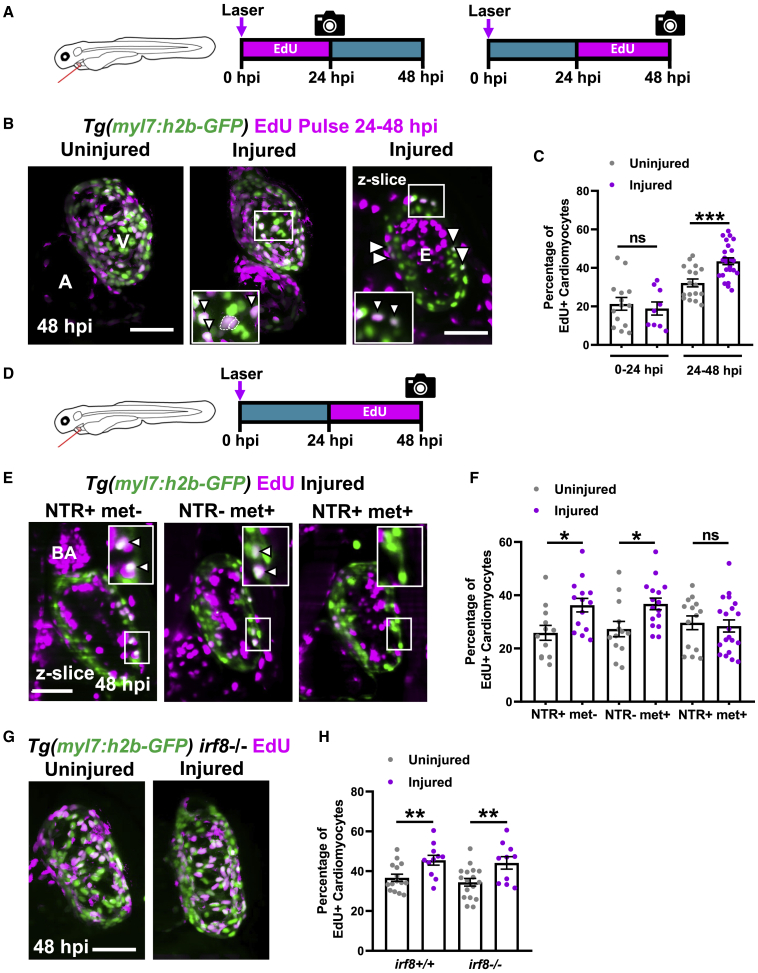
Macrophage ablation abolishes injury-dependent cardiomyocyte proliferation (A) EdU pulse strategy for labeling proliferating cardiomyocytes over 0–24 hpi (left) and 24–48 hpi (right). (B) Images of EdU-stained hearts from *Tg(myl7:h2b-GFP)* larvae at 48 hpi. Non-myocardial EdU signal is excluded post-acquisitionally. A, atrium; v, ventricle; white boxes, zoom panels; white arrowheads, EdU+ cardiomyocyte nuclei; dashed line, outline of dividing cardiomyocyte daughter nuclei. (C) Percentage of ventricular EdU+ cardiomyocytes in uninjured and injured *Tg(myl7:h2b-GFP)* hearts pulsed over 0–24 or 24–48 hpi. ^∗∗∗^ p ≤0.001 unpaired t test, n = 10–25. (D) EdU pulse strategy for labeling proliferating cardiomyocytes over 24–48 hpi in *Tg(myl7:h2b-GFP;csfr1a:NfsB-mCherry)* larvae per standard macrophage ablation groups. (E) Images of EdU-stained hearts from *Tg(myl7:h2b-GFP;csfr1a:NfsB-mCherry)* acquired by LSFM at 48 hpi. White boxes, zoom panels; white arrowheads, EdU+ cardiomyocyte nuclei; BA, bulbous arteriosus. (F) Percentage of ventricular EdU+ cardiomyocytes in uninjured and injured *Tg(myl7:h2b-GFP;csfr1a:NfsB-mCherry)* hearts pulsed over 24–48 hpi. ^∗^p ≤ 0.05 Kruskal-Wallis test and Dunn’s multiple comparison post-hoc test, n = 12–19. (G) Images of uninjured and injured EdU-stained hearts from *irf8*^*−/−*^*Tg(myl7:h2b-GFP)* acquired by LSFM at 48 hpi. Non-myocardial EdU signal is excluded post-acquisition to allow interpretable maximal intensity projections. (H) Percentage of ventricular EdU+ cardiomyocytes in uninjured and injured *irf8*^*+/+*^ and *irf8*^*−/−*^*Tg(myl7:h2b-GFP)* hearts pulsed 24–48 hpi, ^∗∗^ p ≤ 0.01 unpaired t test, n = 10–16. All images are maximum intensity projections of 3D LSFM stacks, unless otherwise stated. Scale bars, 50 μm. Data are represented as mean ± SEM.

To test if cardiomyocyte proliferation increases in response to laser injury, we performed EdU staining in *Tg(myl7:h2b-GFP)* larvae in two experiments. Uninjured and injured larvae were exposed to EdU during 0–24 hpi or alternatively during 24–48 hpi (Figure 4A). Comparison between uninjured and injured hearts revealed no significant difference in the proportion of EdU+ cardiomyocyte nuclei 0–24 hpi (21.3 ± 3.3 versus 18.9 ± 3.4, respectively, n = 10–14) (Figures 4B and 4C). However, over 24–48 hpi there was an organ-wide, 35% increase in the proportion of EdU+ cardiomyocytes in injured hearts relative to uninjured (43.5% ± 1.8% versus 32.2% ± 2.0%, respectively, n = 17–25). Time-lapse *in vivo* imaging of dividing cardiomyocytes showed that nuclear division followed by cytokinesis exclusively gives rise to mononuclear cells, with no obvious hypertrophy (Figure S4A, related to Figure 4).

To understand if macrophages are required for the injury-dependent increase in cardiomyocyte proliferation, EdU was pulsed during the proliferative 24–48 hpi window in the macrophage-less models (Figure 4D). In the metronidazole-nitroreductase ablation model, we found that the percentage of EdU+ cardiomyocytes increased in injured hearts in both the NTR+met− and NTR-met+ control groups, but not in the macrophage-ablated NTR+met+ group (Figures 4E and 4F). This result indicates that macrophages are a requirement for injury-dependent increase in cardiomyocyte proliferation. However, in contrast to the metronidazole-nitroreductase ablation model, analysis of cardiomyocyte proliferation in *irf8*^*−/−*^ mutants revealed that they too significantly increased the percentage of EdU+ cardiomyocytes following injury, comparably with *irf8*^*+/+*^ larvae (Figures 4G and 4H).

To resolve this disparity, we examined more closely the differences between these models. We found, like others (Tsarouchas et al., 2018), that *irf8*^*−/−*^ mutants possess a greater global number of neutrophils than observed in *irf8*^*+/+*^ fish and mount a larger neutrophil response to injury (Figures S4B and S4C, related to Figure 4). Since we do not observe an increased neutrophil response in NTR+met+ larvae, we hypothesized that neutrophils might be compensating for macrophages in *irf8*^*−/−*^ larvae (Figure S4D, related to Figure 4). To test this hypothesis, we inhibited neutrophil recruitment in *irf8*^*−/−*^ larvae using the receptor antagonist “SB225002” that blocks CXCR1/2 activation, a key chemokine receptor for neutrophil migration. CXCR1/2 inhibition successfully lowered the number of recruited neutrophils (2.0 ± 3.4 versus 0.43 ± 0.18) and abolished the injury-associated increase in cardiomyocytes in *irf8*^*−/−*^ (Figures S4E–S4G, related to Figure 4). Taken together, this suggests that macrophages are required for cardiomyocyte proliferation but can be substituted by excess neutrophils.

### Regenerating larval hearts resolve inflammation and enter a reparative stage by 48 hpi

**Figure 5:**
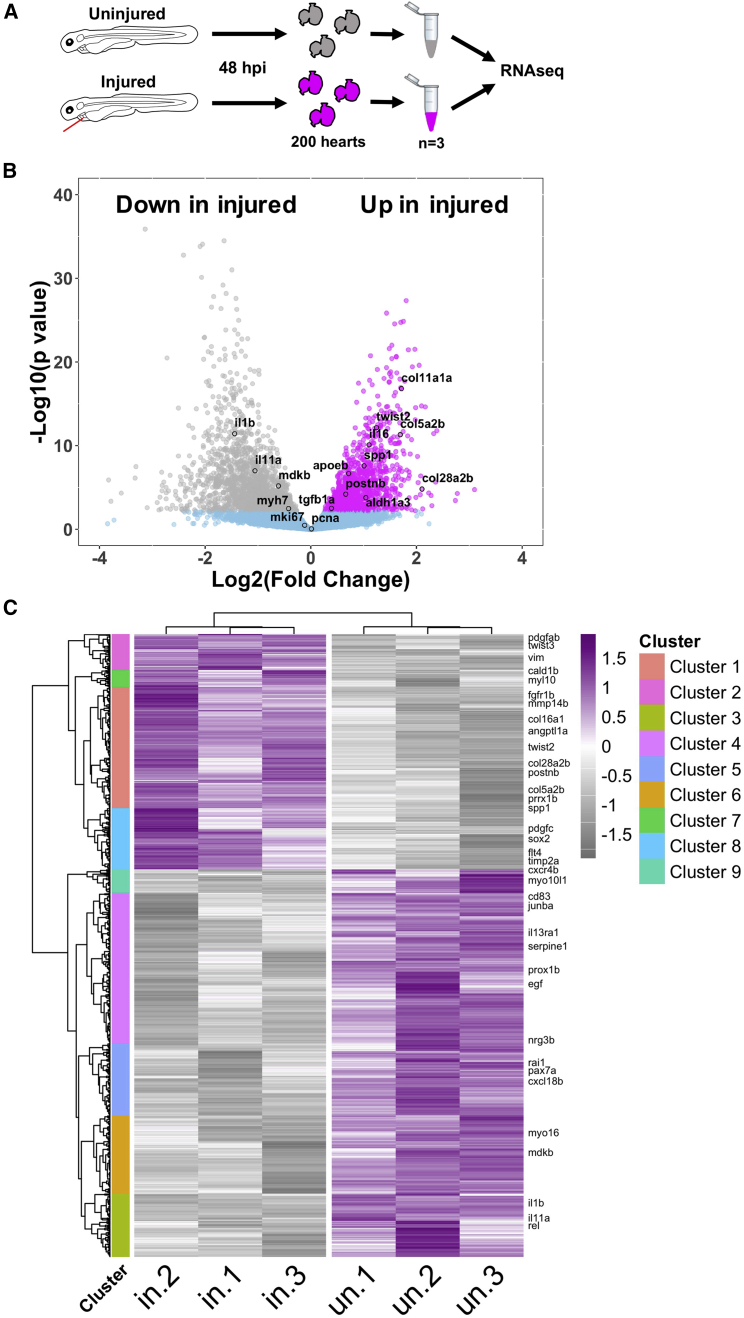
Bulk RNA-seq analysis of larval hearts following injury (A) Extraction of uninjured and injured hearts at 48 hpi and the pooling of 200 hearts per biological replicate for RNA-seq, n = 3. (B) Volcano plot showing the log_2_(fold change) and –log_10_(p value) for transcripts of each detected gene. Genes whose adjusted p values fall below 0.05 are deemed statistically non-significant and colored blue. Genes up regulated in injured hearts are colored magenta and those upregulated in uninjured hearts are colored gray. (C) Heatmap displaying statistically significantly differentially expressed genes with a log_2_(fold change) >0.5. Genes were hierarchically clustered by Pearson correlation with z scaling. Clusters are indicated on the left with their dendrogram. Magenta, high expression; gray, low expression. Genes with relevance to cardiac regeneration are highlighted as annotations on the right of the plot, n = 3.

Next, we sought to understand which biological processes might still be occurring by the final 48 hpi time point of the larval cardiac injury model. We performed RNA-seq on pooled, uninjured, and injured larval hearts at 48 hpi (Figure 5A). We found that 418 genes were upregulated (log_2_ fold change >1) and 1,046 were downregulated in injured hearts. We did not observe differential expression of markers of proliferation such as MCM2, mKi67, and PCNA, suggesting that the proliferation we observe from 24 hpi is concluded by 48 hpi (Figure 5B).

Most inflammatory and M1 markers were either not differentially expressed or were downregulated in injured hearts, such as *il1b* (Figure 5B; Data S1). In contrast, we found injury-associated upregulation of 39 collagen isoforms, several profibrotic genes such as *tgfb1a* and markers of epithelial to mesenchymal transition (EMT) such as *vimentin*. Similarly, hierarchical clustering of differentially expressed genes revealed 9 distinct clusters with cluster 1 being upregulated in injured hearts and enriched in collagens, matrix metalloproteins (MMPs), and fibroblast growth factors (FGFs) (Figure 5C; Data S1). Additionally, cluster 2 contained several EMT genes, cluster 8 genes relating to cell recruitment and lymphangiogenesis, whereas cluster 7 contained several embryonic-associated myosins and myosin binding proteins such as *myl10* and *cald1b*. Clusters 3–6 and 9 were downregulated in injury, cluster 2 was enriched for immune genes, and clusters 4 and 6 for growth factors, with clusters 3 and 5 having no clear identity. Taken together, our RNA-seq results suggest that the inflammatory and proliferative stages are largely concluded by 48 hpi and that a pro-resolving and reparative phase dominates thereafter.

### Cardiac injury induces epicardial activation and *vegfaa* upregulation

Our detailed characterization of the larval laser injury model revealed a macrophage-dependent cardiomyocyte proliferative response occurring at 24–48 hpi. We therefore utilized the rapidity and imaging opportunities offered by the model to investigate the underlying mechanism of the induction of cardiomyocyte proliferation. Epicardial Vegfaa has recently been demonstrated to drive cell cardiomyocyte proliferation in adult zebrafish following cryoinjury, and we hypothesized the same mechanism might drive cardiomyocyte proliferation in the injured larval heart (Karra et al., 2018).

**Figure 6:**
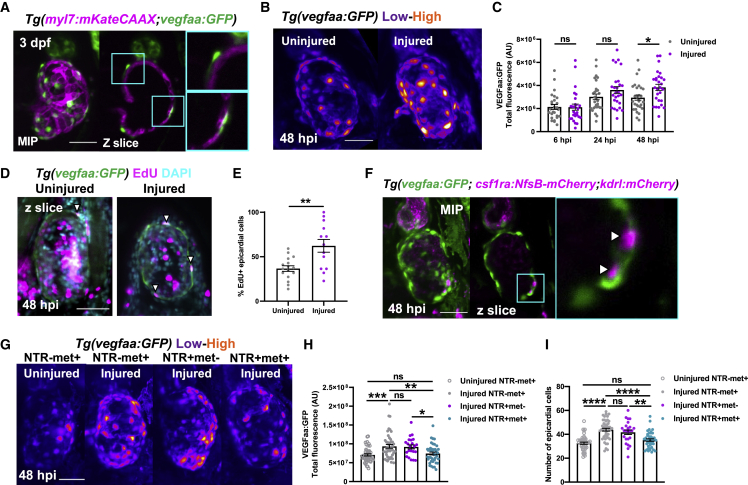
Macrophages stimulate epicardial cell number expansion following cardiac injury (A) Image of an uninjured 3 dpf ventricle from a *Tg(myl7:mKateCAAX;myl7:h2b-GFP)* larva showing vegfaa+ cells (green) overlying myocardium (magenta). Cyan box, zoom panel. (B) Images of uninjured and injured ventricles from *Tg(vegfaa:GFP)* larvae acquired at 48 hpi. “Heat” LUT is applied to highlight the increased intensity of epicardial vegfaa:GFP in injured hearts. (C) Total ventricular *vegfaa:GFP* fluorescence in uninjured and injured hearts over standard injury model time points, n = 28–30. ^∗^ p ≤ 0.05 one-way ANOVA followed by Holms-Sidak’s multiple comparison post-hoc tests. (D and E) Images of uninjured and injured EdU-stained *Tg(vegfaa:GFP)* hearts 48 hpi showing EdU+ epicardial cells (white arrowheads) and the proportion of EdU+ epicardial cells are quantified in (E), n = 13–16. Unpaired t test, ^∗∗^ p ≤ 0.01. (F) Image of a ventricle from a *Tg(vegfaa:GFP;csfr1a:NfsB-mCherry;kdrl:hsa.HRAS-mCherry)* (abbreviated to kdrl:mCherry) larva at 48 hpi showing macrophages in the epicardial-myocardial niche (white arrowheads). Cyan box, zoom panel. (G) Images of uninjured and injured ventricles from *Tg(vegfaa:GFP;csfr1a:NfsB-mCherry)* larvae from metronidazole-nitroreductase macrophage ablation groups at 48 hpi. “Heat” LUT is applied to highlight the increase in the overall fluorescence in injured groups except NTR+met+. Total *vegfaa:GFP* fluorescence (H) and epicardial cell number (I) in uninjured and injured ventricles from *Tg(vegfaa:GFP;csfr1a:NfsB-mCherry)* larvae from metronidazole-nitroreductase macrophage ablation groups at 48 hpi. All images are maximum intensity projections of 3D LSFM stacks. Scale bars, 50 μm, n = 46. ^∗^ p ≤ 0.05, ^∗∗^ p ≤ 0.01, ^∗∗∗^p ≤ 0.001. One-way ANOVA followed by Holms-Sidak’s multiple comparison post-hoc tests. Data are represented as mean ± SEM.

We found robust *vegfaa:GFP* expression specifically in mesothelial cells overlying the myocardium (Figure 6A). Colocalization with established epicardial marker *tcf21* in uninjured *Tg(tcf21:DsRed;vegfaa:GFP)* larvae confirmed these cells to be early epicardium (Figures S5A and S5B, related to Figure 6). Next, we investigated if epicardial *vegfaa:GFP* expression changes following injury by 3D fluorescence intensity analysis of uninjured and regenerating hearts. We found that epicardial *vegfaa:GFP* intensity increased significantly at 48 hpi (Figures 5B and 5C) and that this was organ wide (Figure S5C, related to Figure 6). Interestingly, this was due both to an increase in the number of epicardial cells and their individual intensity (Figures S5D and S5E, related to Figure 6). These data, in combination with an increase proportion of EdU+ epicardial cells in injured hearts (Figures 6D, 6E, and S5F), suggest the epicardium activates and responds to injury by both proliferation and enhanced expression of *vegfaa*.

### Macrophages localize to the epicardial niche and induce the expansion of epicardial cell number

Given that our data showed that macrophage ablation abolishes injury-dependent cardiomyocyte proliferation (Figure 5F), we hypothesized that macrophages might be required for epicardial activation. Indeed, our 3D analysis of macrophage localization following injury showed that recruited macrophages of each subtype do not express *vegfaa* themselves (Figure S5G, related to Figure 6) but can invade the myocardial-epicardial niche and synapse with epicardial cells (Figures 6F and S5H). To test our hypothesis, we ablated macrophages and assessed if epicardial activation still occurred at 48 hpi as before. Following injury, we observed increased *vegfaa:GFP* expression in both macrophage-replete NTR-met+ and NTR+met− groups, but not in macrophage-ablated NTR+met+ hearts (Figures 6G and 6H). Interestingly, macrophage ablation did not affect *vegfaa:GFP* expression per cell but did block the expansion of epicardial cell number following injury (Figures 6I and S5I). Our data therefore strongly suggest that the recruitment of macrophages to the epicardium is essential for subsequent epicardial activation, thus increasing net cardiac *vegfaa* expression.

### Vegfaa is both required and sufficient for cardiomyocyte proliferation in larval zebrafish

**Figure 7:**
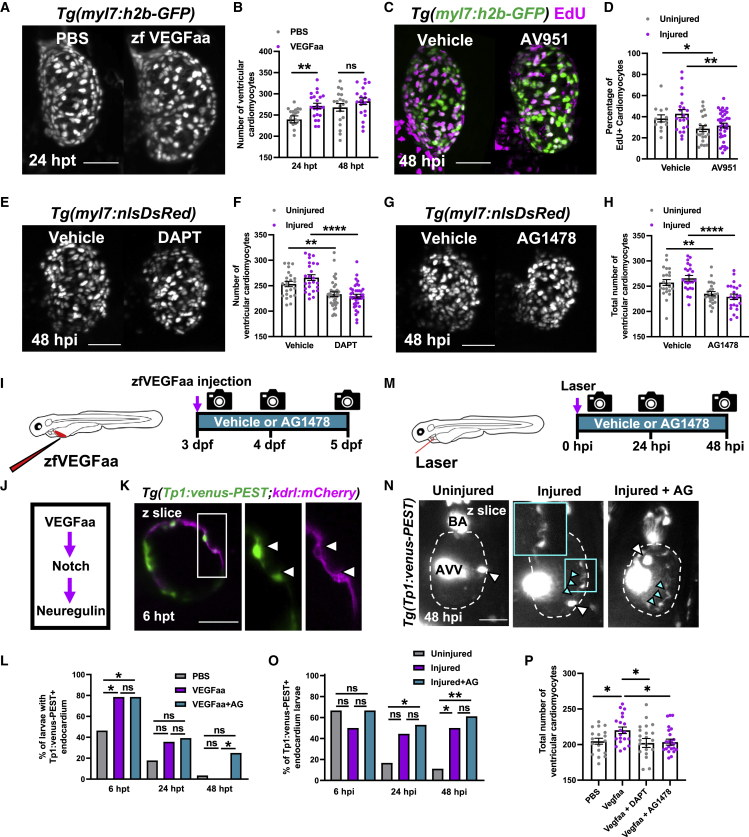
Vegfaa drives cardiomyocyte proliferation by endocardial notch signaling (A) LSFM images of *Tg(myl7:h2b-GFP)* larvae at 24 hpi treated with PBS 0.1% BSA or zfVegfaa 0.1% BSA injection. (B) Ventricular cardiomyocyte number in *Tg(myl7:h2b-GFP)* larvae at 24 and 48 hpi treated with PBS 0.1% BSA or zfVegfaa 0.1% BSA injection, n = 20. Unpaired t test. (C) Images of injured ventricles from *Tg(myl7:h2b-GFP)* larvae, EdU stained and bathed in vehicle or AV951, imaged at 48 hpi. Non-myocardial EdU signal is excluded post-acquisitionally. (D) Percentage of EdU+ cardiomyocyte nuclei from uninjured and injured ventricles from *Tg(myl7:h2b-GFP)* larvae, EdU stained and bathed in vehicle or AV951, n = 13–36. Unpaired t test. (E) Images of injured *Tg(myl7:nlsDsRed)* larvae treated with vehicle or DAPT, acquired at 48 hpi by LSFM. (F) Ventricular cardiomyocyte number in uninjured and injured *Tg(myl7:h2b-GFP)* larvae at 48 hpi treated with vehicle or DAPT, n = 24–40. Unpaired t test. (G) Images of injured *Tg(myl7:nlsDsRed)* larvae treated with vehicle or AG1478, acquired at 48 hpi by LSFM. (H) Ventricular cardiomyocyte number in uninjured and injured *Tg(myl7:h2b-GFP)* larvae at 48 hpi treated with vehicle or AG1478, n = 24. Unpaired t test. (I) Treatment strategy for the injection of uninjured larvae with zfVegfaa and continuous bathing in AG1478 solution. (J) Hypothesized signaling pathway active in uninjured and injured larval hearts driving cardiomyocyte proliferation. (K) LSFM-acquired z plane showing notch expression colocalizing with endocardium in *Tg(Tp1:venus-PEST;kdrl:hsa.HRAS-mCherry)*, abbreviated in the figure to *Tg(Tp1:venus-PEST;kdrl:mCherry)*. AG1478 abbreviated to AG; white box, zoom panel. (L) Proportion of larvae with notch+ endocardium at 6, 24, and 48 hpt following zfVegfaa injection and bathing in AG1478, n = 28. Fisher’s exact test. (M) Treatment strategy for the lasering of larvae and continuous bathing in AG1478 solution. (N) Representative z plane images of uninjured, injured, and injured AG-treated ventricles from *Tg(tp1:venus-PEST)* larvae at 48 hpi. BA, bulbous arteriosus; AVV, atrioventricular valve; white arrowheads, laterally inhibited cardiomyocytes; cyan arrowheads, notch+ endocardium; cyan box, zoom panel. Fisher’s exact test. (O) Proportion of larvae with notch+ endocardium at 6, 24, and 48 hpt following laser injury and bathing in AG1478, n = 18. (P) Cardiomyocyte number at 48 hpi following injection with recombinant Vegfaa and continuous bathing in DAPT or AG1478, n = 22–25. One-way ANOVA followed by Holms-Sidak’s multiple comparison post-hoc tests. All images are maximum intensity projections of 3D LSFM stacks unless otherwise stated. Scale bars, 50 μm. Data are represented as mean ± SEM, ^∗^ p ≤ 0.05, ^∗∗^ p ≤ 0.01, ^∗∗∗^ p ≤ 0.001, and ^∗∗∗∗^p ≤ 0.0001.

To verify if epicardial Vegfaa was driving cardiomyocyte proliferation in larval cardiac regeneration, we first tested if Vegfaa was sufficient to stimulate cardiomyocyte proliferation. Recombinant zebrafish Vegfaa protein (zfVegfaa) was intravenously microinjected into the circulation of 72 hpf *Tg(myl7:h2b-GFP)* larvae and total cardiomyocyte number assessed at 24 and 48 hpt (hours post-treatment). zfVegfaa increased total cardiomyocyte number by 13.3% relative to PBS-injected controls at 24 hpt (Figures 7A and 7B).

To test if VEGF signaling is required for injury-associated cardiomyocyte proliferation, we used a high-affinity, pan-VEGFR receptor antagonist AV951 (Tivozanib) to block VEGF signaling (Chimote et al., 2014). We bathed larvae in 10-nM AV951 over the course of our cardiac injury model, pulsed with EdU over the 24–48 hpi proliferative window, and quantified EdU+ cardiomyocytes at 48 hpi. Interestingly, AV951 decreased the proportion of EdU+ cardiomyocytes in both the uninjured and injured groups (uninjured 38.4 ± 3.4 versus 28.0 ± 3.0 and injured 42.9 ± 3.8 versus 31.6 ± 2.2, n = 13–36) (Figures 7C and 7D). Importantly, treatment with AV951 did not affect lesion closure or recovery of ejection fraction (Figures S6A and S6B, related to Figure 7). Together, these data suggest that VEGF signaling within the heart is driving cardiomyocyte proliferation in the larval heart, both as part of normal development and following cardiac injury.

### Notch and Nrg-ErbB signaling are required for cardiomyocyte proliferation

We next investigated if macrophage-induced epicardial Vegfaa signaling could be interacting with other more established effectors of cardiomyocyte proliferation. Notch and Nrg-ErbB were strong candidates as both are required for adult heart regeneration and cardiomyocyte proliferation in adult zebrafish (Gemberling et al., 2015; Uribe et al., 2018; Honkoop et al., 2019; Zhao et al., 2019). We first verified if these signaling pathways were required for cardiomyocyte proliferation in the larval heart following injury. We laser-injured *Tg(myl7:nlsDsRed)* larvae and bathed them in 100 μM of pan-notch inhibitor DAPT ((N-[N-(3,5-difluorophenacetyl)-l-alanyl]-S-phenylglycine t-butyl ester). DAPT is a gamma secretase inhibitor and has been demonstrated in both zebrafish and *Drosophila* to phenocopy notch mutants (Dovey et al., 2001; Geling et al., 2002; Micchelli et al., 2003). Notch signaling inhibition decreased cardiomyocyte number by ∼8% in uninjured hearts (253.3 ± 5.2 versus 233.4 ± 4.5, n = 25–38) and ∼14% in injured hearts (266.0 ± 5.6 versus 229.5 ± 3.6, n = 25–38) (Figures 7E and 7F).

We repeated this experiment with 1.75 μM ErbB2 antagonist AG1478. Small molecule inhibitor AG1478 selectively inhibits ErbB2, a required co-receptor for ErbB4 dimerization and subsequent neuregulin signal transduction and faithfully phenocopies *erbb2* mutants (Lyons et al., 2005). Interestingly, the results exactly replicated those of the notch signaling inhibition experiment, decreasing cardiomyocyte number by ∼9% in uninjured (257.2 ± 5.8 versus 235.2 ± 4.2) and by ∼13% in injured hearts (265.7 ± 5.5 versus 229.5 ± 5.9) (Figures 7G and 7H). These results confirm that both notch signaling and Nrg-ErbB signaling are required for the expansion of cardiomyocyte number in both uninjured and injured larval hearts.

### Cardiac injury and Vegfaa induce endocardial notch signaling

Given the individual requirement of VEGF, notch, and Nrg-ErbB signaling for cardiomyocyte proliferation in the larval heart, we sought to understand if these signaling components might act in one pathway. Previous studies have demonstrated developmental larval zebrafish heart growth to be activated by cardiac contraction, via endocardial notch > Nrg-ErbB signaling (Samsa et al., 2015; Gálvez-Santisteban et al., 2019). We hypothesized that Vegfaa might be driving cardiomyocyte proliferation by increasing endocardial notch signaling and consequently augmenting this developmental pathway (Figure 7J).

To test if Vegfaa could activate endocardial notch signaling, we utilized the notch signaling reporter line *Tg(Tp1:venus-PEST)* as a readout of cardiac notch signaling. Recombinant zfVegfaa was injected into 3 dpf larvae, and their hearts were analyzed via heart-synchronized LSFM at 6, 24, and 48 hpt (Figure 7I). Furthermore, an additional group of larvae were not only injected with zfVegfaa but also bathed in ErbB2 antagonist AG1478. According to our hypothesized pathway (Figure 7J), we reasoned that zfVegfaa injection should upregulate notch signaling but that inhibition of Nrg-ErbB signaling should be unable to suppress zfVegfaa-induced notch upregulation.

Notch signaling was primarily in the endocardium, colocalizing with endothelial reporter *kdrl:mCherry* but was relatively low intensity and only detectable in a subset of larvae at any given time point (Figure 7K). We found that zfVegfaa injection increased the percentage of larvae with notch+ (Tp1:Venus+) endocardium (46.4% to 78.6%, n = 28) at 6 hpi but not at the 24 and 48 hpi time points (Figure 7L). Furthermore, AG1478 failed to block the increase in the percentage of notch+ hearts following zfVegfaa injection, confirming that Nrg-ErbB signaling was not upstream of *vegfaa* or notch signaling. In fact, zfVegfaa+AG1478-treated larvae had a substantially higher percentage of notch+ hearts at 48 hpi than those treated with zfVegfaa alone (25.0% versus 0%). This is suggestive of a negative feedback mechanism, supporting previous findings of Nrg-ErbB being downstream of notch signaling (Samsa et al., 2015).

To test if this pathway (Figure 7J) acted similarly in injury, we substituted zfVegfaa injection for cardiac injury and repeated the experiment (Figure 7M). Cardiac injury similarly increased the percentage of hearts possessing notch+ endocardium, but this occurred later at 48 hpi (50.0% versus 11.1%, n = 18) (Figures 7N and 7O). As before, AG1478 did not block notch signaling, rather it seemed to enhance it. Although the percentage of notch+ hearts in the injured group did not significantly increase by 24 hpi, injured+AG1478-treated larvae did significantly increase relative to uninjured larvae (52.9% versus 11.1%).

Next, we repeated our zfVegfaa stimulation experiment, this time using *Tg(myl7:h2b-GFP)* larvae and assessed if AG1478 or DAPT blocked Vegfaa-dependent increase in cardiomyocyte number. Both antagonists blocked the response, suggesting that notch and neuregulin signaling act downstream of Vegfaa (Figure 7P).

Finally, we tested if epicardial Vegfaa was likely to be able to reach the endocardium. We measured the diffusion of intravenously injected 500 kDa fluorescent dextran from the blood to the pericardial fluid, finding it to readily traverse the myocardium (Figure S6C, related to Figure 7). Given that zfVegfaa is much smaller (14 kDa), it likely can also diffuse across the myocardium.

Taken together, these results demonstrate that cardiac injury and Vegfaa increase endocardial notch signaling and cardiomyocyte number, providing a mechanism whereby macrophages can trigger cardiomyocyte proliferation via stimulation of epicardial *vegfaa* expression.

## Discussion

In this study, we have presented the first detailed characterization of a larval zebrafish model of heart regeneration, demonstrating the heterogeneity and plasticity of macrophages in cardiac injury and testing the requirement of macrophages for the removal of apoptotic cells, cardiomyocyte proliferation, epicardial activation, and recovery of cardiac structure and function. Furthermore, we demonstrated the utility of the larval cardiac injury model by taking advantage of its *in vivo* cardiac imaging opportunities and amenability to pharmacological intervention to discover a role for macrophages in driving cardiomyocyte proliferation via epicardial activation.

Our examination of macrophages in larval zebrafish cardiac injury suggests that they may faithfully recapitulate the phenotypic complexity and function found in the adult cryoinjury model. As previously shown in adult hearts (Bevan et al., 2020), we detected mpeg1+csf1ra+ and mpeg1+csf1ra− macrophage subsets. We found these cells to have identical recruitment dynamics and no obvious differences in morphology or behavior. Future work should focus on understanding the precise roles of these subsets in cardiac regeneration, in particular mpeg1+csf1ra− macrophages.

In addition to macrophage heterogeneity, we observed macrophage phenotypic plasticity. We used heartbeat-synchronized live imaging to show that both csf1ra+ and mpeg1+ macrophages can convert from tnfa− to tnfa+. Studies examining zebrafish macrophages in spinal cord and tail transection have demonstrated *tnfa* to mark M1-like macrophages, which then transition to M2-like macrophages (Nguyen-Chi et al., 2015). Our success in increasing the percentage of tnfa+ macrophages by canonical M1-polarizing cytokine IFN-γ-rel suggests that early tnfa+ macrophages are indeed proinflammatory. In agreement with findings in the adult cryoinjured heart (Bevan et al., 2020), we found this tnfa+ population of macrophages to be transient, only observed in the early response at 24 hpi. Similarly, our regenerating larval heart RNA-seq dataset showed that by 48 hpi, injured larval hearts downregulate inflammatory cytokines and growth factors but upregulate collagens and reparative cytokines. Furthermore, our finding that a pro-resolving, fibrotic program is activated in injured hearts, despite prior full structural and functional recovery, is in agreement with a recent study showing scar-deficient *runx1*^*−/−*^ zebrafish to undergo successful cardiac regeneration (Koth et al., 2020). Likewise, upregulation of fibrosis-associated genes, such as *prrx1b*, *col11a1a*, and *spp1*, are well documented in adult injury (Bevan et al., 2020; Simões et al., 2020; de Bakker et al., 2021) and would suggest that 48 hpi in larval zebrafish heart regeneration may be comparable with 4–7 dpi in the adult. However, although in the adult this stage is marked by increased cardiomyocyte proliferation, the 48 hpi larval heart has already replaced all lost myocardium. Notably, *vegfaa* is not differentially expressed at 48 hpi, possibly indicating a lag between expression and accumulation of *vegfaa*-driven GFP. It might be that the fibrotic program is concomitantly activated upon resolution of inflammation, irrespective of a requirement for scar tissue.

We used two separate methods to examine the role of macrophages in larval heart regeneration. Interestingly, ablation via csf1ra-driven nitroreductase expression was still able to abolish numbers of csf1ra− macrophages at the injured heart. Possibly this is indicative of a positive-feedback system where csf1ra− macrophage recruitment is dependent on csf1ra+ macrophages. Cell death data acquired by either technique demonstrated that macrophages are required for the timely removal of apoptotic cells following injury. Notably, apoptotic cells are eventually cleared even in the absence of macrophages. The developmental apoptotic cells observed in uninjured *irf8*^*−/−*^ hearts at 4 dpf are also removed and are likely too transient to detect in *irf8*^*+/+*^ hearts. Our live imaging showed that dead cardiomyocytes can be expelled from the myocardium independently of macrophages. This is possibly a consequence of cardiac contraction, although a similar phenomenon is known to occur in neuroepithelium where neurons appear to extrude apoptotic cells out of tissue (Herrgen et al., 2014).

Surprisingly, we found that the absence of macrophages did not affect the structural or functional recovery of the injured larval heart, despite macrophages being required for cardiomyocyte proliferation. This is in contrast to past studies where liposomal clodronate macrophage ablation and CCR2-antagonist inhibition of macrophage recruitment in regenerative neonatal mice, adult zebrafish, and axolotl hearts blocks or delays resolution of the infarct area (Aurora et al., 2014; Leor et al., 2016; Godwin et al., 2017). The contrasting results in the larval heart might simply be a consequence of its small size and low transmural pressure, allowing surviving myocardium to rapidly “knit” back together, independently of cell division. Supporting this, we observed individual cardiomyocytes extending protrusions into the lesion. Previous histological analysis of the border zone in injured adult zebrafish and neonatal mouse hearts has shown cardiomyocytes exhibiting a similar mesenchymal phenotype following Ap-1 and integrin-linked kinase-dependent disassembly of sarcomeres (Morikawa et al., 2015; Beisaw et al., 2020). However, this is the first time this behavior has been verified by time-lapse imaging live in a beating heart.

Studies in adult zebrafish and neonatal mice have shown ablation of macrophages to decrease cardiomyocyte proliferation (Aurora et al., 2014; Lai et al., 2017). However, early revascularization is critical for cardiomyocyte proliferation and is macrophage dependent, calling into question whether macrophages directly induce cardiomyocyte proliferation (Marín-juez et al., 2016; Lai et al., 2017). Larval hearts do not have supporting vasculature (Kaveh et al., 2020); therefore, our finding that cardiomyocyte proliferation is macrophage-dependent suggests that macrophages can facilitate cardiomyocyte proliferation independently of revascularization. Indeed, our data suggest that macrophages are recruited to the epicardial-myocardial niche and induce expansion of epicardial cell numbers, thereby increasing the expression of Vegfaa. This might explain previous findings in developing and injured mouse hearts where yolk-derived and Gata6+ pericardial cavity macrophages are, respectively, recruited to the epicardium (Stevens et al., 2016; Deniset et al., 2019). It is possible that the macrophage-epicardial synapses act to concentrate ligand-receptor pairs as in classical immune synapses (Dustin, 2014). Future studies should seek to identify precisely how macrophages activate epicardium.

Endocardial notch signaling is required for cardiomyocyte proliferation in cryoinjured adults and myocardial growth by downstream Nrg1 in larvae (Zhao et al., 2014, 2019; Samsa et al., 2015). Therefore, our finding that both injury and Vegfaa increase endocardial notch signaling reveals an important mechanism whereby the epicardium can induce cardiomyocyte proliferation. In agreement with previous studies, we showed both notch and Nrg-ErbB signaling to be required for expansion of cardiomyocyte numbers in uninjured and injured hearts (Raya et al., 2003; Gemberling et al., 2015; Zhao et al., 2019). Both signaling pathways appear to be required for the mitogenic response to Vegfaa, suggesting that they are necessary downstream components. Since we found VEGFR inhibition decreased cardiomyocyte proliferation in uninjured larval hearts, it is likely that Vegfaa > notch > Nrg-ErbB is a developmental cardiac growth pathway that is upregulated upon injury and thus might be conserved in mammals. Our discovery that macrophages act upstream of this pathway therefore opens up exciting immunomodulatory opportunities for future therapeutic enhancement of cardiac repair.

### Limitations of the study

This study demonstrates that macrophages are required for epicardial proliferation following cardiac injury. However, the exact mechanism of macrophage-epicardial signaling was not determined. The insights from larval zebrafish should also be tested in mature adult zebrafish and mice to determine their life stage and evolutionary conservation, respectively. Finally, the source and type of neuregulin signaling in larval heart regeneration is still unknown. Future studies are required to understand if Nrg1 or Nrg2a is upregulated and from which cell types.

## STAR★Methods

### Key resources table

**Table:** 

REAGENT or RESOURCE	SOURCE	IDENTIFIER
**Chemicals, peptides, and recombinant proteins**		

Phenylthiourea	Thermo Fisher Scientific	Cat #L06690.09
Tricaine methanesulfonate	Sigma Aldrich	Cat #E10521
Metronidazole	Thermo Fisher Scientific	Cat #210340050
DMSO	Sigma Aldrich	Cat #20-139
Propidium iodide	Thermo Fisher Scientific	Cat #P1304MP
zfIFN-γ-rel (IFN-1.1)	Kingfisher Biotech	Cat #RP1058Z-025
zfVegfaa	Kingfisher Biotech	Cat # RP1040Z-025
Pacific blue 500kDa dextran	Fina Biosolutions	https://www.finabio.net/ (custom made upon request)
AV-951	Stratech Scientific	Cat #A2251-APE
DAPT	Cambridge Bioscience	Cat #CAY13197-10
AG-1478	Cambridge Bioscience	Cat #CAY10010244-10

**Critical commercial assays**		

RedTaq ReadyMix	Sigma Aldrich	Cat #R2523
ApopTag Red In situ kit	MilliporeSigma	Cat #S7165
EdU Imaging Kit with Alexa Fluor 594	Invitrogen	Cat #C10339
RNeasy Plus Micro Kit	Qiagen	Cat #74034

**Deposited data**		

RNA-seq data	This paper (Supplementary file 1)	Raw data are publicly available at Array express: E-MTAB-10860.

**Experimental models: Organisms/strains**		

*Tg(myl7:eGFP)*^*twu*26^	Huang et al. (2003)	N/A
*Tg(mpx:mCherry)*^*uwm*7^	Yoo et al. (2010)	N/A
*Tg(mpeg1:mCherry)*^*gl*23^	Ellett et al. (2011)	N/A
*Tg(mpeg1:eGFP)*^*gl22*^	Ellett et al. (2011)	N/A
*Tg(mpx:GFP)*^*i114*^	Renshaw et al. (2006)	N/A
*Tg(myl7:h2b-GFP)*^*zf52*^	Mickoleit et al. (2014)	N/A
*Tg(myl7:mKateCAAX)*^*SD11*^	Lin et al. (2012)	N/A
*Tg(fms:Gal4.VP16)*^*i186*^	Gray et al. (2011)	N/A
*Tg(UAS-E1b:NfsB-mCherry)*^*c264*^	Davison et al. (2007)	N/A
*Tg(vegfaa:eGFP)*^*PD260*^	Karra et al. (2018)	N/A
*Tg(myl7:nlsDsRed)*^*f2*^	Rottbauer et al. (2002)	N/A
*Tg(TNFa:eGFP)*^*sa43296*^	Nguyen-Chi et al. (2015)	N/A
*Tg(Tp1:venus-PEST)*^*S940*^	Ninov et al. (2012)	N/A
*Tg(kdrl:hsa.HRAS-mCherry)*^*S896*^	Chi et al. (2008)	N/A
*Tg(kdrl:GFP)*^*la116*^	Choi et al. (2007)	N/A
*Tg(tcf21:DsRed)*^*PD37*^	Kikuchi et al. (2011)	N/A
*Tg(myl7:gal4:myl7:GFP*^*)cbg2Tg*^	Mickoleit et al. (2014)	N/A
*irf8*^*st95/st95*^	Shiau et al. (2015)	N/A

**Oligonucleotides**		

Primers for *irf8* genotyping, see method details	This paper	N/A

**Software and algorithms**		

Fiji (ImageJ)	NIH	https://imagej.net/software/fiji/
Imaris	Bitplane	https://imaris.oxinst.com/
R	R core team	https://www.r-project.org/
GraphPad Prism 9.1	GraphPad Software	https://www.graphpad.com/

**Other**		

Neutral red	Thermo Fisher Scientific	Cat #N3246
AvaI restriction enzyme	New England Bioscience	Cat #R0152L

### Resource availability

#### Lead contact

Further information and requests for resources and reagents should be directed to and will be fulfilled by the lead contact, Finnius Bruton (fbruton@ed.ac.uk).

#### Materials availability

This study did not generate new unique reagents. Our RNAseq datasets are publically available at Array Express: E-MTAB-10860.

### Experimental model and subject details

#### Zebrafish husbandry and lines used

Zebrafish husbandry and maintenance was conducted as per standard operating procedures, in accordance with the Animals (Scientific Procedures) Act, 1986 and approved by The University of Edinburgh Animal Welfare and Ethical Review Board in a United Kingdom Home Office-approved establishment. All our experiments were performed on staged zebrafish aged between 3 dpf and 5 dpf. The following transgenic and mutant lines were used: *Tg(myl7:eGFP)*^*twu*26^ (Huang et al., 2003), *Tg(mpx:mCherry)*^*uwm*7^ (Yoo et al., 2010), *Tg(mpeg1:mCherry)*^*gl*23^ (Ellett et al., 2011), *Tg(mpeg1:eGFP)*^*gl22*^ (Ellett et al., 2011), *Tg(mpx:GFP)* ^*i114*^ (Renshaw et al., 2006), *Tg(myl7:h2b-GFP)*^*zf52*^ (Mickoleit et al., 2014), *Tg(myl7:mKateCAAX)*^*SD11*^ (Lin et al., 2012), *Tg(fms:Gal4.VP16)*^*i186*^, referred to as csfr1a:gal4 (Gray et al., 2011), *Tg(UAS-E1b:NfsB-mCherry)*^*c264*^ abbreviated to UAS:NfsB-mCherry (Davison et al., 2007), *Tg(vegfaa:eGFP)*^*PD260*^ (Karra et al., 2018), *Tg(myl7:nlsDsRed)*^*f*2^ (Rottbauer et al., 2002) *Tg(TNFa:eGFP)*^*sa43296*^ (Nguyen-Chi et al., 2015), *Tg(Tp1:venus-PEST)*^*S940*^ (Ninov et al., 2012), *Tg(kdrl:hsa.HRAS-mCherry)*^*S896*^ (Chi et al., 2008), *Tg(kdrl:GFP)*^*la116*^ (Choi et al., 2007)*, Tg(tcf21:DsRed)*^*PD37*^ (Kikuchi et al., 2011), *Tg(myl7:gal4:myl7:GFP*^*)cbg2Tg*^ (Micchelli et al., 2003, Micchelli et al., 2003) and irf8^st95/st95^ (Shiau et al., 2015) referred to as *irf8*^*-/-*^. *Tg(csf1ra:gal4:UAS:NfsB-mCherry)* is abbreviated to csf1ra:NfsB-mCherry throughout the manuscript for simplicity. Adults were day-crossed as appropriate to yield desired combinations of transgenes in embryos. Embryos were treated with 0.003% phenylthiourea (Fisher Scientific) at 7 hpf to prevent pigment formation and therefore enhance image clarity. Embryos and larvae were incubated at 28.5°C in conditioned media/water (6.4 mM KCl, 0.22 mM NaCl, 0.33 mM CaCl_2_·2H_2_O, 0.33 mM MgSO4·7H_2_O) + 0.1% methylene blue (w/v) and imaged at room temperature (23°C) using epifluorescence or light sheet fluorescence microscopy (details below). When necessary, larvae were anesthetized using 40 μg/ml tricaine methanesulfonate (Sigma Aldrich) in conditioned media.

### Method details

#### Cardiac laser injury

A Zeiss Photo Activated Laser Microdissection (PALM) laser system (Zeiss) was used to precisely cause a localised injury at the ventricular apex of anesthetized 72 hpf larvae (Kaveh et al., 2020). Larvae were mounted on a glass slide in 20 μl anesthetized conditioned media and lasered via a 20X objective. Injuries were deemed successful and complete once ventricular contractility decreased, the apex had shrunk, and the myocardial wall had swollen without causing cardiac rupture and subsequent bleeding. A successful cardiac injury results in the portion of dysfunctional tissue losing fluorescent myocardial transgenic fluorescence signal. Uninjured larvae were treated in the same manner up to the point of laser injury, when they were individually transferred into single wells of a 24-well plate and maintained in the same environmental conditions as injured fish.

#### Epifluorescence microscopy

Larvae were mounted laterally in conditioned media on a glass slide and imaged using a Leica M205 FA stereomicroscope with GFP and mCherry filters. For all serial timepoint epifluorescence imaging experiments, number of immune cells on the heart were quantified by manually observing and counting cells moving synchronously with the beating heart. Heart images were acquired using 2X 0.35NA objective.

#### Heart-synchronised light-sheet microscopy

Individual larvae were prepared for light sheet fluorescence microscopy (LSFM) by embedding in 1% low melting-point agarose (ThermoFisher) in anesthetized conditioned media inside FEP tubes (Adtech Polymer Engineering). Agar embedding prevents gradual drift of the embryo in the FEP tube, without causing developmental perturbations during long-term imaging. Larvae were used only once for a time-lapse imaging experiment, and any repeats shown come from distinct individuals. Larvae were mounted head down such that the heart faces toward both illumination and imaging objectives to improve image clarity. All LSFM experiments were performed at room temperature (23°C). Camera exposure times ranged from 5-15 ms, laser excitation power was 11mW and scans were performed at 3-5 minute intervals. Brightfield images acquired at 80 fps were analysed in real-time to enable optically-gated acquisition of fluorescence z slices at a set phase of cardiac contraction, usually mid diastole. The setup of our custom-built LSFM system has been previously reported in detail (Taylor et al., 2019).

#### Metronidazole-nitroreductase macrophage ablation model

To selectively ablate macrophages prior to cardiac injury, embryos were incubated as previously described until 48 hpf and then treated as follows. Embryos were carefully dechorionated at 48 hpf and screened based on fluorescence and split into groups appropriate to the experiment, for example larvae were always split into csf1ra:gal4;UAS:NfsB-mCherry+ and csf1ra:gal4;UAS:NfsB-mCherry-. Embryos were then transferred to either conditioned water or a 0.5mM metronidazole (Thermo Fisher Scientific) solution, both solutions also contained 0.003% phenylthiourea (Thermo Fisher Scientific) and 0.2% DMSO (Sigma Aldrich). Larvae were then incubated in these solutions in the dark at 28. 5°C for 24 hours prior to injury at 72 hpf. Larvae were then removed from the metronidazole solution and vehicle solution and placed in fresh conditioned water + 0.003% phenylthiourea for the remainder of the experiment. As shown in Figure 2, this is sufficient to ablate macrophages prior to injury and completely block subsequent macrophage recruitment to the injured heart.

#### Neutral red staining

Larvae were incubated at 72 hpf in 5μg/mL neutral red (Thermo Fisher Scientific) in conditioned water for 5 hours in the dark at 28.5°C. Larvae were then washed twice for 5 minutes in conditioned water, anaesthetised with 40 μg/ml tricaine methanesulfonate and imaged by brightfield microscopy on a Leica M205 FA stereomicroscope.

#### Genotyping of *irf8*^*-/-*^ mutants

Adult (>30 dpf) zebrafish arising from heterozygous *irf8* mutant incrosses were anaesthetised in 40 μg/ml tricaine methanesulfonate and a lobe of caudal fin removed by scalpel. After clipping, fins were digested to extract DNA using 10mg/ml Prot K, incubated at 65oC for 1 hour. This incubation ends with 15 minutes at 95°C to denature the Proteinase K. A section of irf8 flanking the mutation locus was then amplified from the extracted DNA by PCR using Forward -ACATAAGGCGTAGAGATTGGACG and Reverse -GAAACATAGTGCGGTCCTCATCC primers and REDTaq® ReadyMix™ PCR Reaction Mix. The PCR product was then digested for 30 minutes at 37°C using AVA1 restriction enzyme (New England Bioscience) and the product run on a 2% agarose gel. WT = AvaI digest site is present = PCR product is cleaved to give two bands with sizes of approximately 200 and 100 bp. irf8 ^-/-^ = AvaI digest site is absent due to mutation = PCR product is not cut. A single band is observed with a size of 286 bp. irf8 ^+/-^ = Three bands as above.

#### Microinjection recombinant proteins and intravital stains

Microinjections were performed on larvae at 72 hpf using a Narishige IM-300 Microinjector and pulled thin wall glass capillaries (Harvard Apparatus), administered under anaesthesia by intravenous microinjection through the cardiac sinus venosus (SV) that drains the common cardinal vein (CCV). An injection volume of 1 nL was used for all intravenous injections to minimise disruption to blood volume.

For propidium iodide intravital staining, 1nL 100μg/ml propidium iodide in DPBS was injected immediately following injury at 0.5 hpi. Larvae were then immediately imaged by heartbeat-synchronised light-sheet microscopy at 1 hpi. Injection of recombinant zfIFN-γ-rel (IFN-1.1) (Kingfisher Bioscience) was administered as a single 1nL 132nM dose at 72 hpf. Lyophilised IFN- γ-rel was reconstituted in PBS + 0.1% BSA (carrier protein) and PBS + 0.1% BSA was used as the vehicle control solution. Injections of recombinant zfVegfaa (Kingfisher Bioscience) were administered as single 1nL 0.25 ug/ul doses at 72 hpf (protein reconstituted as above).

Pacific blue tagged 500 kDa dextran (Fina Biosolutions) (dissolved at 5% w/v in DPBS) was injected at 1nL into injured larvae 2 hpi and the heart and pericardial fluid immediately imaged by heartbeat-synchronised light-sheet microscopy.

#### Histological staining

To detect cell death at the injured ventricle, whole-mount larval TUNEL staining was performed. Larvae were fixed in 4% PFA for 30 minutes and transferred to 1:10 dilution of PBS. Larvae were subsequently digested in 1 μg/ml Proteinase K for 1 hour. Larvae were re-fixed in 4% PFA for 20 minutes and subsequently washed in PBT. TUNEL staining was performed using ApopTag Red In situ kit (MilliporeSigma) to label apoptotic cells, as described previously (Kaveh et al., 2020). Stained hearts were imaged using LSFM.

EdU staining was performed by incubating larvae in 1 mM EdU (5-ethynyl-2′-deoxyuridine) (Abcam) in 1 % DMSO (Sigma Aldrich) in conditioned water + 0.003% phenylthiourea (Thermo Fisher Scientific) for 24 hours beginning either at 0 hpi or 24 hpi depending on the experiment. Larvae were incubated at 28.5°C in the dark. Larvae were then fixed for 2 hours at room temperature in 4% PFA, permeabilised in permeabilisation solution (PBS-Triton-X 0.1% + 1% Tween + 1% DMSO) and pericardium punctured using a glass microinjection needle (further improving permeability). Larvae were then washed twice in PBS-3% BSA and incubated for 2 hours at room temperature in CLICK reaction mixture from Click-iT™ EdU Imaging Kit with Alexa Fluor™ 594 (Invitrogen) made according to manufacturers’ instructions. Larvae were finally washed once in PBS-3%BSA and twice in PBS-0.1% tween and imaged by LSFM.

#### Pharmacological inhibition of larval signalling

To inhibit VEGF signalling, larvae were bathed in pan-VEGFR antagonist AV951/Tivozanib (Stratech Scientific) 0-48 hpi. AV951 was dissolved in 0.1% DMSO in conditioned water + 0.003% phenylthiourea to make a 10 nM solution, with just 0.1% DMSO in conditioned water + 0.003% phenylthiourea becoming the vehicle control. In order to pulse larvae with EdU, the original solution was replaced fresh solution, with the addition of 1mM EdU at 1% DMSO.

To inhibit notch signalling, larvae were bathed in gamma secretase inhibitor DAPT (Cambridge Bioscience) 0-48 hpi. DAPT was dissolved in 0.2% DMSO in conditioned water + 0.003% phenylthiourea to make a 100μM solution, with just 0.2% DMSO in conditioned water + 0.003% phenylthiourea becoming the vehicle control. Note, DAPT must be dissolved in DMSO prior to the addition of water to prevent precipitation.

In order to inhibit neuregulin-ERBB signalling, the ErBB2 antagonist AG1478 was used. Larvae were bathed in 1.75 μM AG1478 (Cambridge Bioscience) dissolved in 0.25% DMSO in conditioned water + 0.003% phenylthiourea over 0-48 hpi.

#### Extraction of larval hearts and RNA extraction

Following laser injury at 72 hpf *Tg(myl7:gal4::GFP;UAS:mRFP)* larvae were incubated at 28.5°C in conditioned media/water + 0.1% methylene blue (w/v) + 0.003% phenylthiourea. At 48 hpi uninjured and injured larvae were given an overdose of tricaine at 400 μg/ml, following which hearts were extracted. We adapted the protocol of Burns and MacRae (2006) to increase the yield of heart retrieval from ∼50% to ∼70%. Briefly, ∼30 larvae were placed in 2mL eppendorf tubes, the conditioned water drained and replaced with ice cold Leibovitz's L-15 Medium supplemented with 10% FCS. A 19-gauge needle coupled to a 5mL syringe was used to shear the larvae by aspiration and therefore dissociate hearts from the rest of the larva. The lysate was then inspected by epifluorescence microscopy and mRFP+ hearts and collected to be kept on ice. Hearts were then digested for 10 minutes at 4°C in protease solution (5 mM CaCl2,10 mg/ml B. Licheniformis protease, 125 U/mL DNase I in 1x PBS) with occasional aspiration to aid digestion, RNA was then extracted using a RNeasy Plus Micro Kit (Qiagen) following direct lysis with RLT lysis buffer according to manufacturer’s instructions. RNA concentration was measured by Qubit and integrity by Bioanalyser. RIN score for all samples ranged between 9.6-10.

### Quantification and statistical analysis

#### Heart lesion size quantification

Larval hearts expressing the transgene *myl7:GFP* were imaged by heartbeat-synchronised light-sheet imaging as described above. Exposure was kept consistent at 10ms, along with z slice spacing (1 μm), and heart contraction phase was locked to mid diastole for all larvae. Z stacks were surface rendered in IMARIS (Bitplane) based on absolute intensity, and software-suggested segmentation and rendering parameters. Lesion area, visualised as a render-free hole in the myocardium, was then traced around manually and lesion area quantified in FIJI (National Institutes of Health) (Schindelin et al., 2012).

#### Ventricular ejection fraction analysis

Larval hearts of *Tg(myl7:GFP)* larvae were imaged at 80 fps in brightfield using a Leica M205 FA epifluorescence stereomicroscope, to capture when the ventricle was in diastole and systole. The ventricular area in diastole and systole was measured manually in FIJI and ventricular ejection fraction calculated using the formula 100 X [(Diastolic Area – Systolic Area)/Diastolic Area] (Matrone et al., 2013). Ventricular ejection fraction by area was then converted to ejection fraction by volume using the formula ‘Ejection fraction by area x 2.33 = Ejection fraction by volume’. Over the small range of ejection fractions that occur in larval hearts, the relationship can be considered to approximate to a linear one.

#### Quantification of cell number by image analysis

To quantify the number of cardiomyocytes in *Tg(myl7:h2b-GFP)* and *Tg(myl7:nlsDsRed)* larval hearts, and also epicardial cells in *Tg(vegfaa:GFP) and Tg*(*tcf21:DsRed*) hearts, z stacks of hearts acquired by LSFM were imported into FIJI and nuclei counted using the plugin Trackmate. Briefly, key segmentation parameters ‘Estimated blob diameter’=5.5, ‘Threshold’=0.9 were taken as a starting point, and optimised manually per experiment until all nuclei or cell bodies are counted successfully. Nuclei are easily automatically detected, as are the thick central cell body of epicardial cells. The heart atrium is excluded manually by x coordinate filtering and ventricular cardiomyocytes are then automatically counted by the plug in.

In order to automatically quantify the percentage of EdU+ ventricular cardiomyocytes in *Tg(myl7:h2b-GFP)* larval hearts, a custom FIJI macro was written to exclude non-cardiomyocyte EdU signal. This is necessary as cardiomyocytes have a much lower turnover rate than surrounding cells in the pericardium, endocardium and blood and so represent a minority of EdU+ cells. Briefly, the Bersen segmentation method was used to mask areas of GFP fluorescence per z slice and these masks subsequently applied as a crop RoI to EdU signal in the 641 nm colour channel of RGB images. Slices were then reassembled and merged into maximum intensity projections, where the FIJI (Schindelin et al., 2012) Trackmate plugin was used to count both the total number of GFP+ cardiomyocyte nuclei and EdU+ cardiomyocyte nuclei. This quantification then allowed the percentage of EdU+ cardiomycytes to be calculated in an unbiased way per larval heart.

#### Quantification of notch signalling by image analysis

In order to objectively identify whether the hearts from *Tg(Tp1:venus-PEST)* larvae possessed venus signal in the endocardium above that of background, and were therefore ‘notch+’, the following approach was used. Treatment groups were blinded to the analyser, and z stacks opened in FIJI. The automatic brightness and contrast function was used to objectively enhance the signal in the heart, and the clear interface between the granular autofluorescence of the chamber blood and the smooth autofluorescence of the myocardium searched for venus expression. The distinctive morphology and location of endothelium allowed for unambiguous identification of venus+ status.

#### RNAseq analysis

RNA was sequenced by Genewiz, Leipzig, Germany using Illumina NovaSeq, PE 2x150. Genewiz also used deseq2 package in R to evaluate sequencing quality, trim reads, map to the *Danio rerio* genome and generate gene counts/hits. Sequence reads were trimmed using Trimmomatic v.0.36. The trimmed reads were mapped to the *Danio rerio* GRCz10.89 reference genome available on ENSEMBL using the STAR aligner v.2.5.2b. Unique gene hit counts were calculated by using featureCounts from the Subread package v.1.5.2. Only unique reads that fell within exon regions were counted. The Wald test was used to generate p-values and log_2_ fold changes. A gene ontology analysis was performed on the statistically significant set of genes by implementing the software GeneSCF v.1.1-p2. The zfin GO list was used to cluster the set of genes based on their biological processes and determine their statistical significance. The volcano plot was generated by a custom R script and heatmap constructed using the pHeatmap package. For the heatmap z scaled log_2_(Reads) were clustered via Pearson correlation and clusters thresholded based on the resulting dendrogram. The heatmap was generated using the pHeatmap function in R.

#### Statistics

Graphs and statistics were curated in GraphPad Prism 9.1 software (GraphPad Software). Data were analysed by student t-test, one-way ANOVA or two-way ANOVA followed by an appropriate multiple comparison *post hoc* test. All statistical tests, *p-value*s, precision measures and *n* numbers used are given in figure legends, p<0.05 was deemed significant in all experiments.

## Data Availability

• Bulk RNA-seq data have been deposited at ArrayExpress and are publicly available as of the date of publication. Accession numbers are listed in the key resources table. Microscopy data reported in this paper will be shared by the lead contact upon request. • This paper does not report original code. • Any additional information required to reanalyze the data reported in this paper is available from the lead contact upon request.

## Supplemental information

Document S1. Figures S1–S6

Data S1. Bulk RNA-sequencing normalized gene read counts per group and genes present per cluster, related to Figure 5

Document S2. Article plus supplemental information
