# Supplementary material for: Macrophages trigger cardiomyocyte proliferation by increasing epicardial vegfaa expression during larval zebrafish heart regeneration
Source: Dev Cell. 2022 Jun 20;57(12):1512–1528.e5. doi: 10.1016/j.devcel.2022.05.014 (PMC9616726; doi:10.1016/j.devcel.2022.05.014)
Supplement: Document S1. Figures S1–S6 [file mmc1.pdf]

Developmental Cell, Volume *57*

Supplemental information

Macrophages trigger cardiomyocyte proliferation by increasing epicardial *vegfaa* expression during larval zebrafish heart regeneration

Finnius A. Bruton, Aryan Kaveh, Katherine M. Ross-Stewart, Gianfranco Matrone, Magdalena E.M. Oremek, Emmanouil G. Solomonidis, Carl S. Tucker, John J. Mullins, Christopher D. Lucas, Mairi Brittan, Jonathan M. Taylor, Adriano G. Rossi, and Martin A. Denvir

Supplementary figures

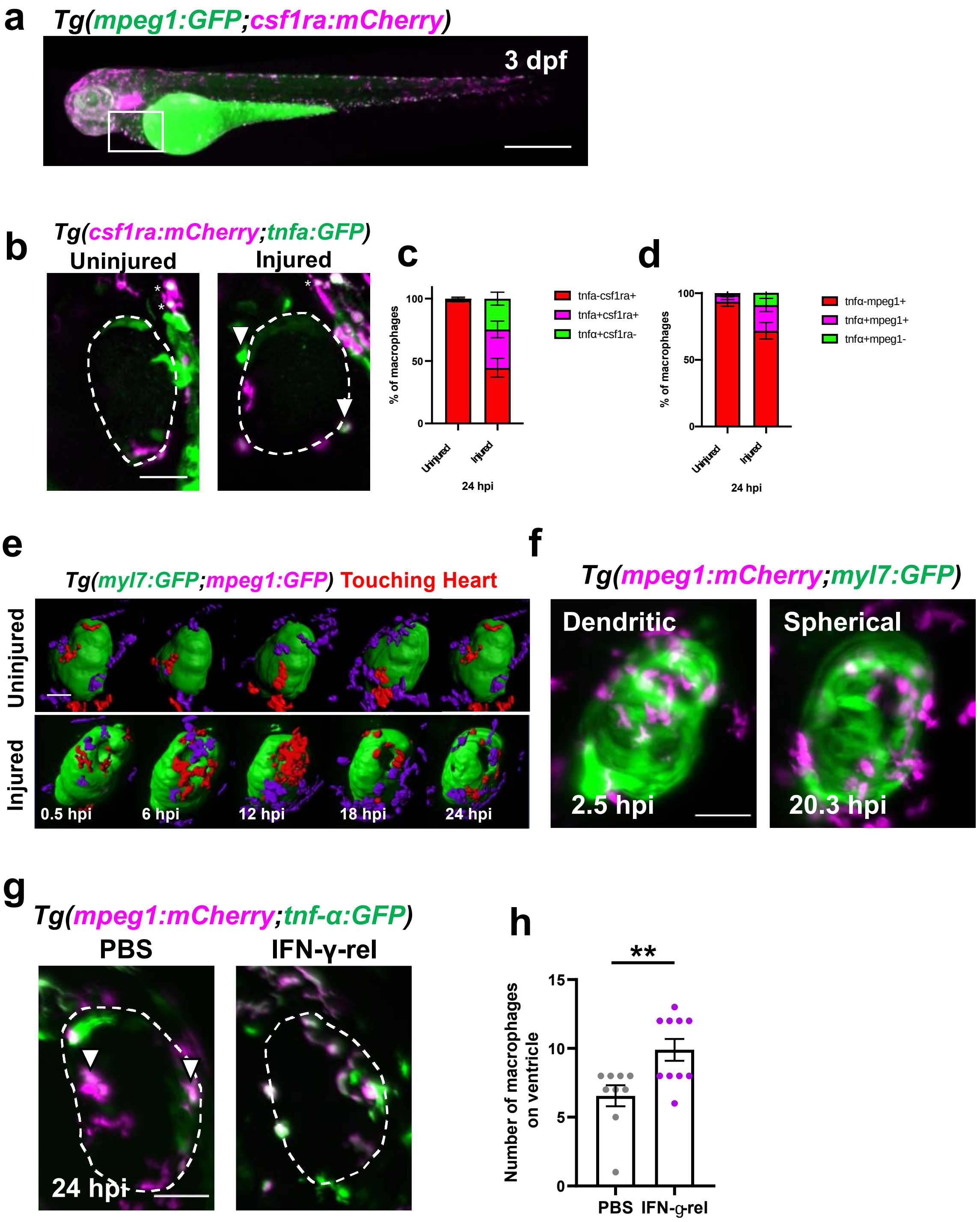

Supplementary figure 1: Cardiac macrophage phenotype in larval zebrafish is plastic and can be polarised to tnfa+ by IFN-γ-rel, related to Figure 1.

(a) Representative epifluorescence image of a 3 dpf *Tg(mpeg1:GFP;csf1ra:NfsB-mCherry)* abbreviated to *Tg(mpeg1:GFP;csf1ra:mCherry)* in the figure, showing an anterior-posterior polarity in macrophage expression of csf1ra (higher proportion of

anterior macrophages were csf1ra+). White box = indicated pericardial area. Scale bar = 500μm. (b) Representative LSFM acquired images of uninjured and injured *Tg(csf1ra:mCherry;tnfa:GFP)* hearts at 24 hpi. White arrowheads = co-expressing macrophages, asterisks = autofluorescent green nuclei, scale bar = 50μm. (c) Quantification of the proportion of macrophages that are tnfa+csf1ra+, tnfa-csf1ra+ and tnfa+csf1ra- on hearts in uninjured and injured larvae at 24 hpi. (d) Quantification of the proportion of macrophages that are tnfa-mpeg1+, tnfa+mpeg1+ and tnfa+mpeg1- on hearts in uninjured and injured larvae at 24 hpi. (e) Time-lapse timepoints of *Tg(myl7:GFP;mpeg1:mCherry)* hearts acquired by heartbeat-synchronised LSFM, surface rendered and colour-coded to show myocardium in green, macrophages on the heart in red and macrophages elsewhere in purple. Macrophages can be seen to change from stellate to rounded over time following injury. Scale bar = 50μm. (f) LSFM acquired images (MIPs) of an injured *Tg(myl7:GFP;mpeg1:mCherry)* heart at the timepoints indicated at the figure showing macrophage shape transition from dendritic to spherical, scale bar = 50μm. (g) Representative images of hearts from *Tg(mpeg1:mCherry;tnfa:GFP)* larvae at 24 hpi injected with PBS or IFN-γ-rel. White dashed line = outline of the ventricle; and white arrowheads = tnfa+mpeg1+ macrophages. Scale bar = 50μm. (h) Quantification of the number of macrophages on the injured ventricle at 24 hpi after injection at 0 hpi with PBS or IFN-γ-rel. Data are represented as mean +/- SEM.

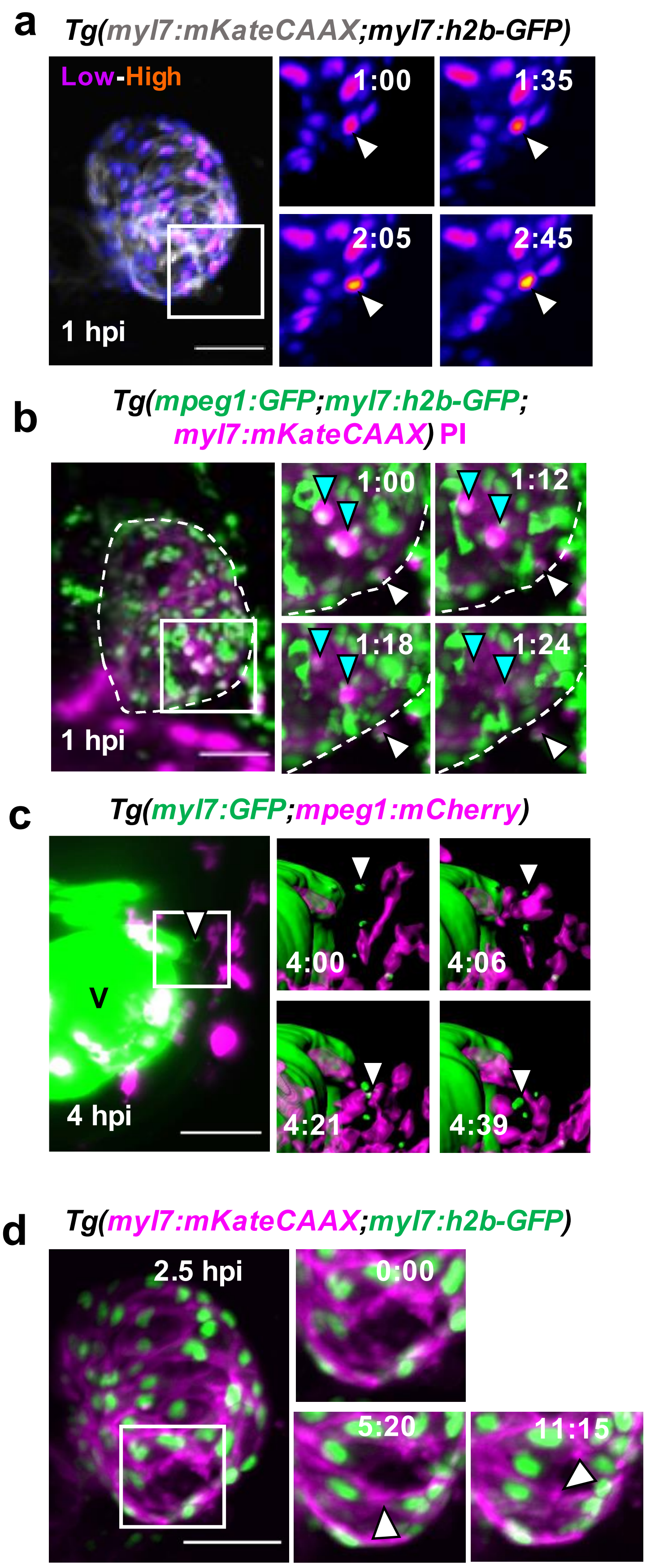
a
Tg(myl7:mKateCAAX;myl7:h2b-GFP)
Low-High
1 hpi
1:00
1:35
2:05
2:45
b
Tg(mpeg1:GFP;myl7:h2b-GFP;
myl7:mKateCAAX) PI
1 hpi
1:00
1:12
1:18
1:24
c
Tg(myl7:GFP;mpeg1:mCherry)
V
4 hpi
4:00
4:06
4:21
4:39
d
Tg(myl7:mKateCAAX;myl7:h2b-GFP)
2.5 hpi
0:00
5:20
11:15

Supplementary figure 2: Heartbeat-synchronised lightsheet-acquired time-lapse stills, related to Figures 2 & 3.

(a) Time-lapse stills of injured *Tg(myl7:h2b-GFP;myl7:mKateCAAX)* ventricles imaged from 1 hpi. GFP intensity show by heat LUT, white arrowhead = apoptotic cardiomyocyte/condensing nucleus, white box = zoom panel. (b) Time-lapse stills of injured *Tg(myl7:h2b-GFP;myl7:mKateCAAX;mpeg1:GFP)* ventricles imaged from 1 hpi by heart-synchronised light-sheet imaging. Round GFP^{low} = cardiomyocyte nuclei and stellate GFP^{high} =macrophages. Cyan arrowheads = Necrotic cardiomyocyte nuclei and white arrowheads = expelled necrotic cardiomyocyte, white box = zoom panel. (c) Time-lapse stills of an injured *Tg(myl7:GFP;mpeg1:mCherry)* ventricle from 4 hpi where the full size panel has high gain in the GFP channel to highlight GFP^{low} myocardial debris and zoom panels (area indicated by white box) are surface rendered to highlight removal of myocardium (green) by macrophages (magenta). V = high gain ventricle, white arrowhead = myocardial debris. (d) Time-lapse stills of an injured *Tg(myl7:mKateCAAX;myl7:h2b-GFP)* ventricle from 2.5 hpi. White box = zoom panel, white arrowheads = cell-cell junctions

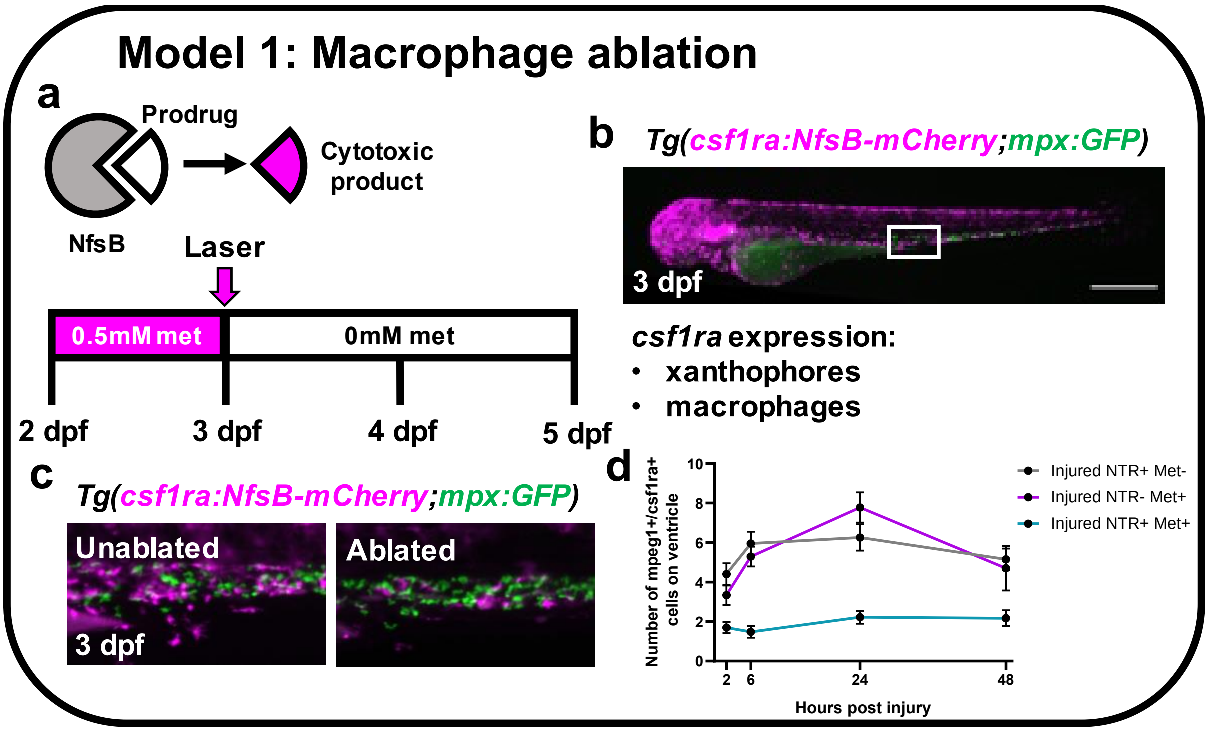

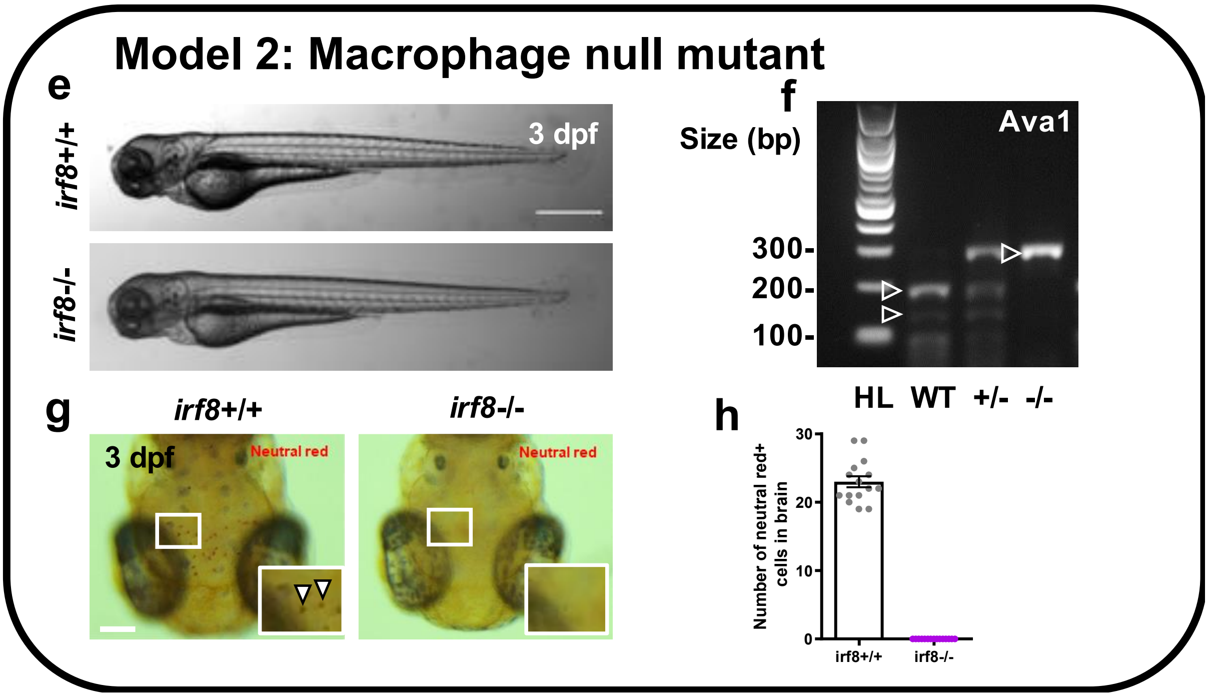

Supplementary figure 3: Macrophages can be pharmacologically ablated or developmentally blocked genetically, related to Figure 2.

(a) Schematic illustrating how nitroreductase enzyme 'NfsB' catabolises prodrug 'metronidazole' to form a cytotoxic biproduct. (b) Representative epifluorescence image of a *Tg(csf1ra:NfsB-mCherry;mpx:GFP*) 3 dpf larva (abbreviated to *Tg(csf1ra:mCherry;mpx:GFP)* in all panels), white box = caudal haematopoietic tissue,

magenta = macrophages and green = neutrophils (CHT) (c) Representative images of ablated and unablated macrophages in the CHT, size and location indicated in (b)) in *Tg(csf1ra:mCherry;mpx:GFP)* 3dpf larvae. Macrophages are ablated and only apoptotic bodies remain but not neutrophils are unaffected. (d) Quantification of macrophages at standard timepoints, marked by either mpeg1 or csfr1a on the injured ventricle in each of the NTR=metronidazole ablation model's treatment groups NTR+Met-, NTR-Met+ and NTR+Met+. Macrophage ablation can be seen to abolish the macrophage response (e) Representative brightfield images of *irf8*$^{+/+}$ and *irf8*$^{-/-}$ larvae at 3 dpf. (f) Representative 1% agarose gel displaying Ava1 restriction digest band pattern for WT, *irf8* heterozygous and homozygous mutants. (g) Representative dorsal view brightfield image of 3 dpf larval heads stained with neutral red vital dye with white zoom panel highlighting stained macrophages (microglia) (red) in *irf8+/+* but not *irf8*$^{-/-}$ larvae. (h) Quantification of the number of neutral red positive stained cells (macrophages/microglia) in larval brains of *irf8*$^{+/+}$ and *irf8*$^{-/-}$ at 3 dpf showing *irf8*$^{-/-}$ larvae to be macrophage-null. Scale bar = 500μm (b & e), 100μm (g).

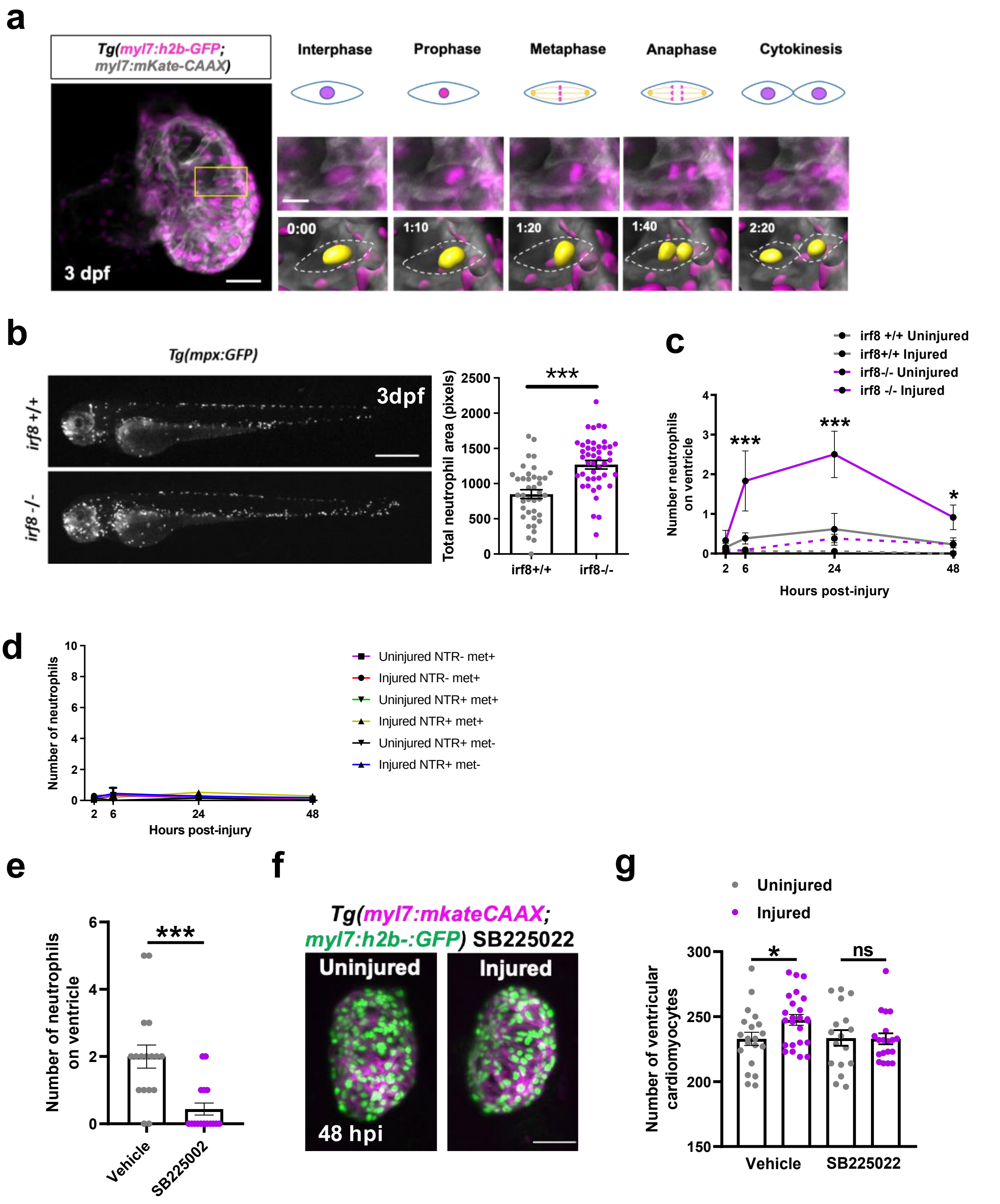

Supplementary figure 4: *irf8*$^{-/-}$ larvae have a larger neutrophil response to cardiac injury than *irf8*$^{+/+}$, related to Figure 4.

(a) Representative timepoint images from heartbeat-synchronised LSFM time-lapse of a laser-injured 3 dpf *Tg(myl7:h2b-GFP;myl7:mKateCAAX)* larva showing an example of each phase of complete cell division of a single cardiomyocyte, typical of larval hearts. Yellow box = zoom panel; left scale bar = 30 μm; right scale bar = 10 μm. Timestamps post-injury indicated in figure. (b) Representative whole larva

epifluorescence image of *irf8*$^{-/-}$ and *irf8*$^{+/+}$ *Tg(mpx:GFP)* larvae showing *irf8*$^{-/-}$ to have greater global neutrophil numbers (scale bar = 500 μm), quantified in the graph (right). ***$p \leq 0.001$. t test, n=39-46. (c) Quantification of neutrophil numbers at the ventricle in uninjured and injured *irf8*$^{+/+}$ and *irf8*$^{-/-}$ larvae at the standard laser-injury model timepoints, showing *irf8*$^{-/-}$ larvae to have a significantly greater neutrophil response. n=17-25. (d) Quantification of neutrophil numbers at the ventricle in uninjured and injured NTR-met+, NTR+met+ and NTR+met- larvae at the standard laser-injury model timepoints. All metronidazole-nitroreductase treatment groups to have a minimal neutrophil response and therefore no neutrophil compensation in the macrophage ablated group NTR+met+, n=17-24. (e) Quantification of the number of recruited neutrophils at the injured ventricle in at 24 hpi in *Tg(myl7:h2b-GFP;myl7:mKateCAAX)* larvae bathed in vehicle or SB225002 from -2 to +24 hpi showing SB225002 to significantly reduce neutrophil number, n=17. (f) Representative light-sheet acquired images of uninjured and injured *irf8*$^{-/-}$ *Tg(myl7:h2b-GFP;myl7:mKateCAAX)* ventricles at 48 hpi following treatment with SB225002 from -2 to +24 hpi, scale bar = 50 μm. (g) Quantification of ventricular cardiomyocyte number in uninjured and injured *irf8*$^{-/-}$ *Tg(myl7:h2b-GFP;myl7:mKateCAAX)* ventricles at 48 hpi following treatment with vehicle or SB225002 -2 to 24 hpi, n=17-20, * $p \leq 0.05$ t test. Data are represented as mean +/- SEM.

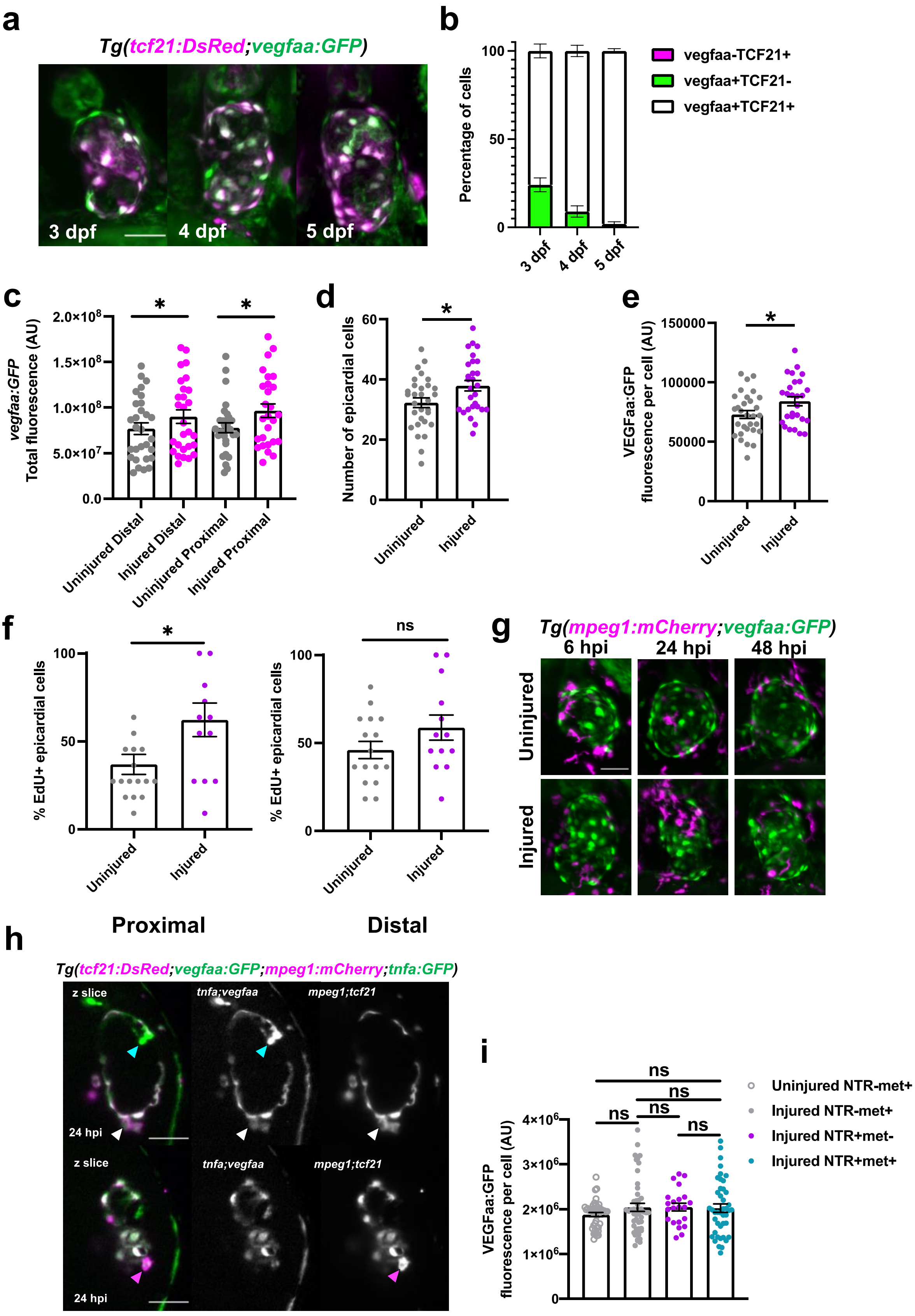

a
Tg(tcf21:DsRed;vegfaa:GFP)
3 dpf
4 dpf
5 dpf
b
Percentage of cells
vegfaa-TCF21+
vegfaa+TCF21-
vegfaa+TCF21+
c
vegfaa:GFP Total fluorescence (AU)
Uninjured Distal
Injured Distal
Uninjured Proximal
Injured Proximal
d
Number of epicardial cells
Uninjured
Injured
e
VEGFaa:GFP fluorescence per cell (AU)
f
% EdU+ epicardial cells
ns
Proximal
Distal
g
Tg(mpeg1:mCherry;vegfaa:GFP)
6 hpi
24 hpi
48 hpi
Uninjured
Injured
h
Tg(tcf21:DsRed;vegfaa:GFP;mpeg1:mCherry;tnfa:GFP)
z slice
tnfa;vegfaa
mpeg1;tcf21
24 hpi
i
VEGFaa:GFP fluorescence per cell (AU)
Uninjured NTR-met+
Injured NTR-met+
Injured NTR+met-
Injured NTR+met+

Supplementary figure 5: *vegfaa:GFP* expression does not colocalize with macrophages following larval heart injury, related to Figure 6.

(a) Representative images of 3, 4 and 5 dpf ventricles from a *Tg(tcf21:DsRed;vegfaa:GFP)* larvae acquired by LSFM, showing high colocalization of *vegfaa* expression with epicardial marker *tcf21*. White arrowheads = heterogenous marker expression and white box = zoom panel. These proportions are quantified in (b), n=7. (c) Quantification of total GFP fluorescence in uninjured and injured *Tg(vegfaa:GFP)* hearts at 48 hpi by region. Proximal = half of the heart nearest to the lesion, Distal = half of the heart furthest from the lesion, n= 30. T-test, *$p \leq 0.05$. (d) Quantification of the number of epicardial cells, as marked by *vegfaa:GFP*, on injured ventricles at 48 hpi in uninjured and injured larvae, n=30. *$p \leq 0.05$ t test (e) Quantification of the average *vegfaa:GFP* expression of epicardial cells per cell, on injured ventricles at 48 hpi in uninjured and injured larvae, n=30. *$p \leq 0.05$ t test. (f) Region specific (proximal and distal) quantification of the proportion of EdU+ *vegfaa*+ epicardial cells in uninjured and injured *Tg(vegfaa:GFP)* hearts at 48 hpi, n=13-16. *$p \leq 0.05$ t-test. (g) Representative LSFM image of an injured *Tg(vegfaa:GFP;mpeg1:mCherry)* heart 24 hpi showing *vegfaa:GFP* expression only in the epicardium and not in macrophages, scale bar = 100μm. (h) Representative LSFM-acquired images of macrophage-epicardial synapsing by mpeg1+tnfa- and mpeg1+tnfa+ macrophages in *Tg(tcf21:DsRed;vegfaa:GFP; mpeg1:mCherry;tnfa:GFP)* hearts at 24 hpi. (i) Quantification of average *vegfaa:GFP* fluorescence per cell in metronidazole-nitroreductase ablation model groups at 48 hpi, n=22-44. One-way ANOVA followed by Holm-Sidak's multiple comparisons Post-hoc test. All scale bars = 50 μm. Data are represented as mean +/- SEM.

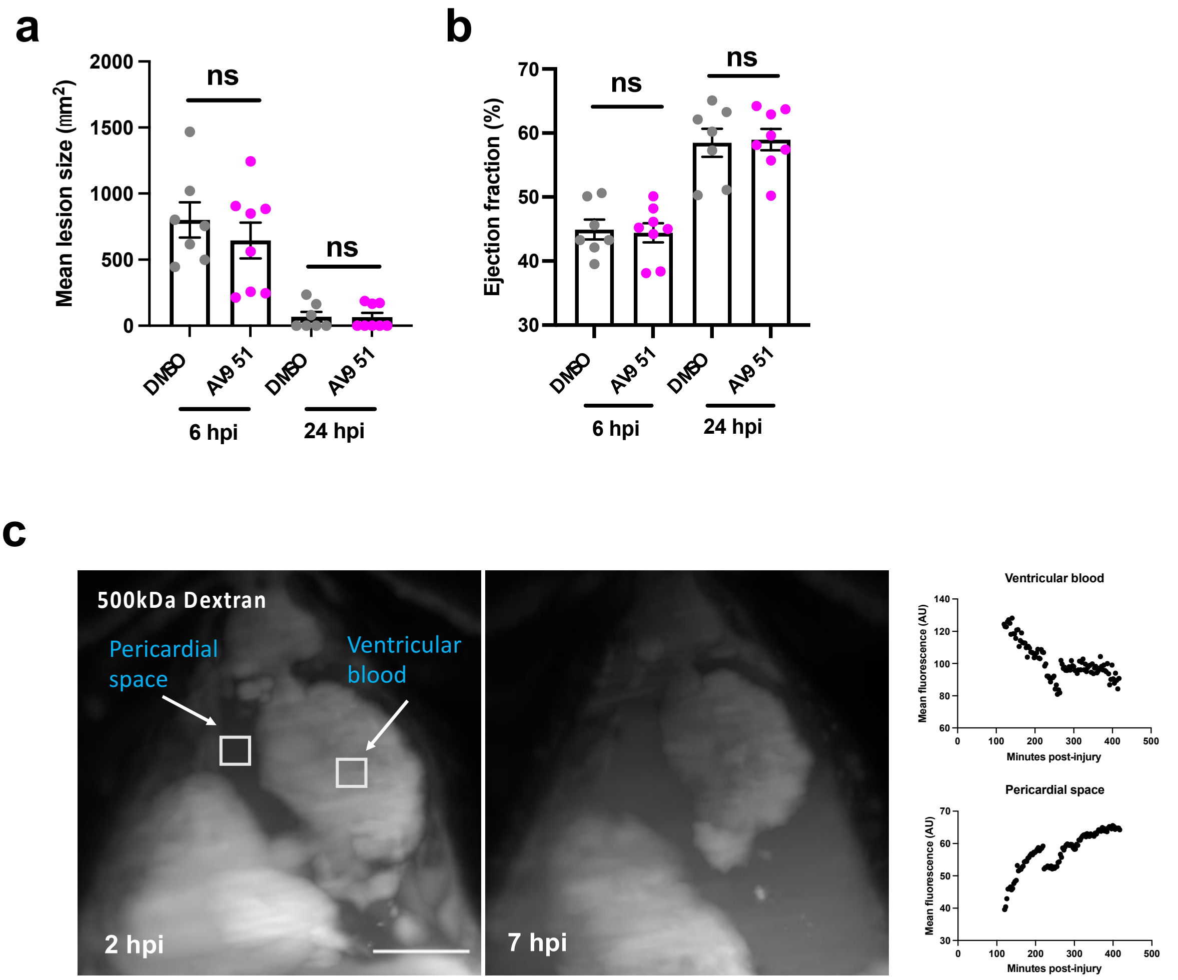

Supplementary figure 6: Vegfaa is able to diffuse across the wall of the larval heart, related to Figures 3 & 7.

(a) Quantification of mean lesion size (μm) in injured *Tg(myl7:GFP)* larvae at 6 hpi and 24 hpi when continuously bathed in vehicle (DMSO) or AV951. (b) Quantification of ejection fraction (%) in injured *Tg(myl7:GFP)* larvae at 6 hpi and 24 hpi when continuously bathed in vehicle (DMSO) or AV951. (c) Representative LSFM acquired images (left) of larval hearts following injection with 500 kDa fluorescently labelled dextran, shown at 2 hpi and 7 hpi. The fluorescent signal of the pericardial space and ventricular blood (white boxes) was quantified every 5 minutes, plotted (right) and can be seen to equilibrate. Scale bar = 50 μm. Data are represented as mean +/- SEM.
